# Integrated pancancer analysis reveals the oncogene characteristics and prognostic value of DIP2B in breast cancer

**DOI:** 10.1186/s12885-023-10751-3

**Published:** 2023-03-31

**Authors:** Chengyang Song, Fangjian Shang, Wei Tu, Xiaodan Liu

**Affiliations:** 1 Department of Thoracic and Cardiovascular Surgery, the Fourth Affiliated Hospital of China Medical University, Shenyang, China; 2 Department of General Surgery, the Fourth Affiliated Hospital of China Medical University, Shenyang, China

**Keywords:** DIP2B, Oncogene, Pancancer, Breast cancer, Her-2 positive, Prognosis, Immune microenvironment, Immunosuppression, Immunotherapy

## Abstract

**Background:**

Disco-interaction protein 2 homologue B (DIP2B) plays an important role in DNA methylation. There have been many reports on DIP2B in various diseases, but neither the diagnostic value nor the prognostic value of DIP2B across cancer types has been deeply explored.

**Methods:**

The expression levels of DIP2B in 33 cancer types were analysed based on data sets from The Cancer Genome Atlas (TCGA) and the Genotype-Tissue Expression (GTEx) database. The relationships of DIP2B expression with immune cell infiltration and immune-related gene expression were studied via the CIBERSORT, ESTIMATE and TISIDB tools. Gene set variation analysis (GSVA) was performed to identify pathways related to DIP2B. DIP2B knockdown by siRNA was performed in breast cancer cell lines to investigate the effect on proliferation, apoptosis and migration. The relationships of DIP2B expression with clinicopathological features and prognosis were analysed based on immunohistochemistry.

**Results:**

DIP2B was highly expressed in 26 of 33 cancer types and was significantly associated with poor overall survival (OS) in breast invasive carcinoma (BRCA), mesothelioma and chromophobe renal cell carcinoma (each *P* < 0.05). DIP2B showed a negative correlation with the immune score, the infiltration levels of key immune killer cells (CD8 + T cells, activated NK cells and plasma cells), and the expression of major histocompatibility complex–related genes and chemokine-related genes in BRCA. Subtype analysis showed that DIP2B expression was associated with poor OS in Her-2 + BRCA patients (*P* < 0.05). DIP2B showed a negative correlation with immune killer cell infiltration and immune regulatory genes in BRCA subtypes. In BRCA, the GSVA results revealed that genes correlating positively with DIP2B were enriched in cancer-related pathways (PI3K-AKT) and cell-cycle-related pathways (MITOTIC_SPINDLE, G2M_CHECKPOINT and E2F_TARGETS), while genes correlating negatively with DIP2B were enriched in DNA_REPAIR. Knockdown of the DIP2B gene induced a reduction in proliferation and migration and an increase in apoptosis in breast cancer cell lines. DIP2B expression was associated with lymph node metastasis and poor histological grade in BRCA according to immunohistochemistry (each *P* < 0.05). DIP2B expression predicted reduced disease-free survival and OS in BRCA patients (each *P* < 0.05), especially those with the Her-2 + subtype (*P* = 0.023 and *P* = 0.069).

**Conclusions:**

DIP2B may be a prognostic biomarker for BRCA, especially for the Her-2 + subtype. DIP2B is associated with a “cold” tumour immune microenvironment in BRCA and might serve as a future target for immunotherapy.

**Supplementary Information:**

The online version contains supplementary material available at 10.1186/s12885-023-10751-3.

## Introduction

Cancer treatment strategies, including surgery, chemotherapy, radiotherapy, targeted therapy and immunotherapy, have shown success in the clinic. However, due to drug resistance, side effects, or other problems, the prognosis of patients is still unsatisfactory. Consequently, finding new biomarkers or therapeutic targets for cancer diagnosis and treatment is urgently needed.

Disco-interaction protein 2 homologue B (DIP2B), a member of the DIP2 family, was first identified in *Drosophila* as a binding partner of Disconnected and conserved from *Caenorhabditis elegans* to humans [1]. The DIP2B protein contains a binding site for the transcriptional regulator DNA methyltransferase 1 associated protein 1 (DAMP1) and binding sites for AMP. These sites suggest that the DIP2B protein may participate in DNA methylation [2]. Adlat et al*.* reported that DIP2B-deficient mouse embryonic lung fibroblasts showed a reduction in cell proliferation and migration and an increase in apoptosis [3]. Sah R. K. et al*.* found that DIP2B plays a role in the cell cycle, cell division, and G2/M phase transition and is essential for lung development [4]. Interestingly, DIP2B has been identified as a potential susceptibility gene associated with colorectal cancer [5, 6], and the epigenetic modifications of DIP2B mediated by miRNAs may be implicated in the metastasis of cervical squamous cell carcinomas [7]. However, the diagnostic value and prognostic value of DIP2B across cancers have not been deeply explored.

Immunotherapy has emerged as one of the most promising anticancer therapies. However, only a proportion of patients benefit from immune checkpoint inhibitors (ICIs) [8, 9]. The tumour microenvironment has been recognized as an important participant in tumour progression [10]. Numerous preclinical studies have shown that T-cell infiltration in the tumour microenvironment (TME) is the basic mechanism for blocking immune checkpoints and that the baseline T-cell density in tumours correlates with the response to ICIs in melanoma and other solid tumours [11–13]. The roles of macrophages, B cells and other immune cells are also under evaluation in immunotherapy [14]. A recent study showed that one-allele knockout of DIP2B in mice significantly promoted the growth and metastasis of subcutaneously implanted tumours, decreased tumour cell apoptosis and reduced immune cell infiltration in tumours, most likely by altering the immune system by reducing macrophage and cytotoxic T-cell infiltration into the tumour microenvironment [15]. However, the effect of DIP2B expression on immune infiltration in primary cancer tissues is still unknown.

Our study comprehensively analysed the relationship between DIP2B expression and prognosis in 33 cancer types by bioinformatics. The correlation between DIP2B expression and the tumour immune microenvironment was discussed. DIP2B expression in BRCA was further explored. The biological function of DIP2B was studied in breast cancer cell lines. The relation of clinicopathological features of BRCA patients with DIP2B expression was analysed by immunohistochemistry. The flow chart of the present study is shown in Supplemental Fig. 1.

## Materials and methods

### TCGA data acquisition and difference analysis

As the largest cancer gene information database, The Cancer Genome Atlas (TCGA) database (https://portal.gdc.cancer.gov/) stores data on gene expression, copy number variation, single nucleotide polymorphisms (SNPs) and other variables. The original mRNA expression data and SNP data of 33 different tumour types were downloaded from the Genotype Tissue Expression (GTEx) database (https://commonfund.nih.gov/GTEx) and the TCGA and corrected to calculate the difference in gene expression between cancer and normal tissues for different cancer types. Tumour cell line data was downloaded from the Cancer Cell Line Encyclopedia (CCLE) database (https://portals.broadinstitute.org/ccle/), and the expression levels of genes in these tumour cell lines were analysed on the basis of the tissue source. In addition, the correlation between gene expression and tumour stage was studied.

### Prognostic correlation analysis

The overall survival (OS) data of TCGA cohorts were downloaded from the Xena database to further study the relationship between gene expression and prognosis. The Kaplan‒Meier (KM) method was adopted for survival analysis of each cancer type, and survival analysis was performed via the "survival" and "survminer" packages. Additionally, Cox analysis was performed with the "survival" and "forestplot" packages to explore the relationship between gene expression and survival.

### Immune cell infiltration analysis

The CIBERSORT algorithm was used to analyse the RNA-seq data of patients with 33 cancer types in different subgroups, aiming to infer the relative proportion of immune infiltrating cells and analyse the correlation between gene expression and immune cell infiltration level. The tumour purity, stromal score and immune score of tumour samples were estimated by the ESTIMATE algorithm [16]. Furthermore, the potential relationship between gene expression and immunoregulatory factors (including major histocompatibility complex-related genes, chemokine-related genes, immunostimulator-related genes, immunoinhibitor-related genes and chemokine receptor-related genes) was explored through the TISIDB website.

### Gene Set Variation Analysis (GSVA)

GSVA is a nonparametric and unsupervised method to evaluate the enrichment of gene sets at the transcriptome level. GSVA comprehensively scores the gene set of interest, transforms the gene level change data into the pathway level change data, and then judges the biological function of the sample. In this study, gene sets were downloaded from the Molecular Signatures Database (v7.0). The GSVA algorithm was used to comprehensively score each gene set to evaluate potential changes in biological functions of different samples.

### Tumour Mutation Burden (TMB) and microsatellite instability (MSI) data analysis

TMB is defined as the total number of somatic gene coding errors, base substitutions, insertions or deletions detected per million bases. In this study, TMB was defined by calculating the variation frequency and variation/exon length of each tumour sample and dividing the number of nonsynonymous mutation sites based on the total length of the protein coding region. Microsatellite instability (MSI) values for each TCGA patient were derived from previously published studies [17].

### Tissue specimens

Breast cancer tissue specimens were obtained from 120 patients who underwent radical surgery at the Fourth Affiliated Hospital of China Medical University (Shenyang, China) from January 2008 to December 2012. Clinical data were retrospectively collected from medical records. Prognosis data were collected from medical records or telephone follow-up of patients or relatives of the patients. The exclusion criteria of patients were as follows: (1) unclear histological grade; (2) use of neoadjuvant chemotherapy or radiotherapy before surgery; and (3) incomplete tumour resection (R1 or R2). Breast cancer staging was based on the American Joint Committee on Cancer (AJCC) Cancer Staging Manual (8th, 2017).

### Cell culture

The human breast cancer cell lines MCF-7, T47D, SK-BR-3 and MDA-MB-231 were obtained from the Cell Bank of the Chinese Academy of Science (Shanghai, China) in October 2022. MCF-7 cells were cultured in MEM containing 10% foetal bovine serum (HyClone, Logan, UT, USA) and 0.01 mg/ml insulin. T47D cells were cultured in RPMI-1640 medium containing 10% foetal bovine serum (HyClone, Logan, UT, USA) and 0.2 U/ml insulin. SK-BR-3 cells were cultured in McCoy’s 5A medium containing 10% foetal bovine serum (HyClone, Logan, UT, USA). MDA-MB-231 cells were cultured in Leibovitz’s L-15 medium containing 10% foetal bovine serum (HyClone, Logan, UT, USA). All the cell lines were cultured in 1% penicillin/streptomycin in a humidified atmosphere at 37 °C with 5% CO_2_. Cells were grown on sterilized glass Petri dishes and detached for subculture using 0.25% trypsin (Gibco, Carlsbad, CA, USA). All cell lines were authenticated using STR profiling, and all experiments were performed with mycoplasma-free cells.

### SiRNA and transfection

Human DIP2B siRNA (sense strand, 5’- GGCAUGUUUGCGAAUGUAAdTdT -3’; antisense strand, 5’- UUACAUUCGCAAACAUGCCdTdT -3’) and negative control siRNA (nonsilencing siRNA) were purchased from Beijing DingGuoChangSheng Biotech Co., Ltd. (Beijing, China). Nontransfected cells were used as the blank control group. Breast cancer cells were seeded in 6-well culture plates at a density of 3 × 10^5^ cells/well. After incubation overnight, the cells were transiently transfected with DIP2B-siRNA (2.5 μg/well) or the nonsilencing siRNA using Lipofectamine 2000 (Invitrogen, Carlsbad, CA, USA) according to the manufacturer’s instructions. The ability of DIP2B-siRNA to inhibit DIP2B mRNA and protein expression was analysed by real-time PCR and Western blotting.

### Real-time PCR analysis

RNA was isolated from breast cancer cells using TRIzol reagent (Invitrogen) according to the manufacturers’ instructions. After verification of purity and concentration, the RNA was transcribed into cDNA using the cDNA Synthesis kit (Invitrogen). The cDNA was subjected to real-time PCR using the SYBR Green PCR Supermix kit (Invitrogen) with the Rotor gene-3000 instrument (Corbett). Reactions were performed in 20 μL volumes with 1 μL cDNA. Primer sequences for DIP2B were 5’-GGCAGATGACCCCTGTGAAA-3’ and 5’-TTCCCTGTCATGTCCAGTGC-3’. The primer sequences used for GAPDH were 5’-GAAAGCCTGCCGGTGACTAA-3’ and 5’-AGGAAAAGCATCACCCGGAG-3’. The PCR protocol was as follows: 95 °C for 2 min and then 45 cycles of 95 °C for 15 s and 60 °C for 30 s. Relative expression was calculated using the 2^−ΔΔCt^ method with GAPDH serving as a reference gene for normalization.

### Western blot analysis

Total protein was extracted from breast cancer cells in lysis buffer containing 50 mM Tris–HCl (pH 8.0), 150 mM NaCl, 0.5% Nonidet P40, 0.5% sodium deoxycholate, and phenylmethylsulfonyl fluoride (PMSF; Sigma‒Aldrich Chemicals, St Louis, MO, USA). The protein concentration was determined by BCA assay. One hundred micrograms of protein per sample was separated by SDS‒PAGE and transferred onto nitrocellulose membranes. The membranes were rinsed in Tris-buffered saline with Tween-20 (TBS-T) and blocked in 5% nonfat dry milk/TBS-T prior to incubation in rabbit polyclonal antibodies against DIP2B (1:500; bs-14332R, BIOSS, Beijing, China) overnight at 4℃. Following incubation in the primary antibody, the membranes were washed and then incubated in a goat anti-rabbit antibody solution (1:3000, 65–6120, Thermo Fisher Scientific, USA). The immunoreactive protein bands were detected by chemiluminescence (Thermo, Waltham, MA, USA). GAPDH was used as a loading control (1:2000, MA5-15,738, Thermo Fisher Scientific, USA).

### CCK-8 assay

The CCK-8 assay was used to assess cell proliferation after transfection with DIP2B-siRNA. The cells were seeded in 96-well plates. At 24, 48, and 72 h after transfection, cell viability was determined using a Cell Counting Kit-8 (Dojindo, Japan) by measuring the optical density at 450 nm (OD450) with a microplate reader (Bio-Rad Laboratories, USA).

### Flow cytometry

The effect of DIP2B-siRNA on the apoptosis of the cells was determined by flow cytometry. Cells of each group at 24 h post transfection were trypsinized and collected by centrifugation at 800 r/min for 5 min. They were then incubated at room temperature for 15 min with 0.5 mL of binding buffer and 1 μL Annexin V-FITC from the Annexin V-FITC apoptosis detection kit (Merck, Darmstadt, Germany). After that, the cells were resuspended in 0.5 mL fresh binding buffer containing 5 μL propidium iodide (PI), and then, apoptosis was detected by a FACSCalibur flow cytometer (BD Biosciences, San Jose, CA, USA).

### Scratch assay

A scratch assay was used to evaluate breast cancer cell migration before and after DIP2B siRNA transfection. Breast cancer cells (1 × 10^6^/well) were seeded in 6-well plates and cultured overnight. Then, a scratch was carefully draw across a layer of confluent breast cancer cells using a 200 µL sterile pipette tip, and cell debris was discarded; the remaining cells were washed with culture medium twice and cultured again for up to 24 h with serum-reduced medium containing 1% FBS. Images of the plates were taken under a microscope at 0 h, and the closure of the gap was measured at 24 h.

### Immunohistochemistry

Formalin-fixed and paraffin-embedded tissue specimens were cut into 3-μm-thick sections. To detect DIP2B, the sections were deparaffinized in xylene and rehydrated in a graded alcohol series. The sections were subsequently heated in citrate buffer (pH 6.0) for 30 min at 93 °C in a microwave oven for antigen retrieval and then incubated in 20% normal serum for 50 min at room temperature. Sections were incubated with an anti-DIP2B antibody 1:400 (bs-14332R, BIOSS, Beijing, China) at 4 °C overnight. Sections were incubated with phosphate buffered saline (PBS) instead of the primary antibody as a negative control. The next day, sections were incubated with an appropriate secondary antibody (ab-6112; Abcam, UK) for 30 min at room temperature. After that, the sections were incubated with a 3,3’-diaminobenzidine solution for up to 2 min to allow colour development. The stained tissue sections were reviewed and diagnosed independently by two experienced pathologists who were blinded to the sample type. DIP2B was evaluated as negative (-), weakly positive ( +), moderately positive (+ +) or strongly positive (+ + +). Negative and weakly positive staining were considered to indicate low expression, and moderately positive and strongly positive staining were considered to indicate high expression. If a disagreement occurred, the stained sections were re-evaluated to reach a consensus.

### Statistical analysis

R language (version 4.0) and SPSS (version 26.0) were used for statistical analyses. Pearson’s chi-squared (χ^2^) test or Fisher’s exact test was used to analyse the association between DIP2B expression and clinicopathological features. Hazard ratios (HRs) and 95% confidence intervals were calculated using univariate survival analysis. Kaplan‒Meier analysis was carried out to compare the survival of patients grouped by DIP2B expression (high versus low). Independent sample t tests were used for cytology experiments. A *P* value less than 0.05 was considered to indicate statistical significance.

## Results

### DIP2B analysis across cancers

#### Pancancer analysis of DIP2B gene expression

**Fig. 1:**
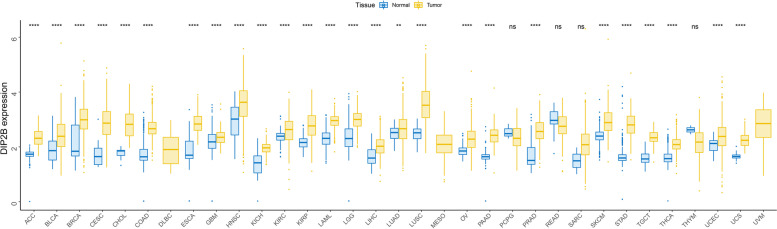
The expression of DIP2B in TCGA and GTEx pancancer tissues. ns = not significant (P > 0.05); *P < 0.05; **P < 0.01; ***P < 0.001; ****P < 0.0001

**Fig. 2:**
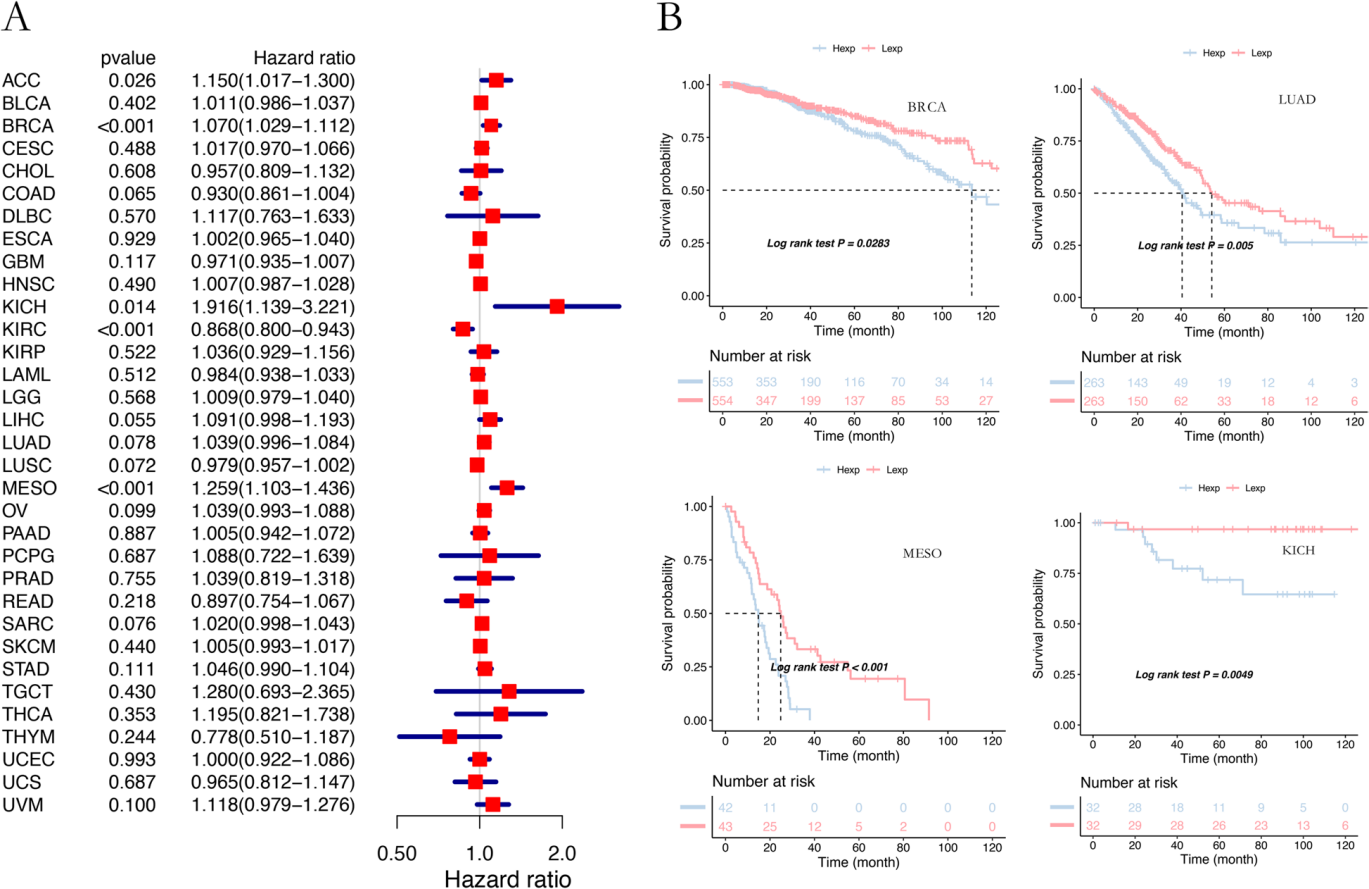
Analysis of the correlation of DIP2B with overall survival (OS) across cancers. **A** Univariate Cox regression analysis of the relationship between DIP2B and OS. HR > 1 indicates a risk factor, and HR < 1 indicates a protective factor. The expression of DIP2B was closely related to worse OS in ACC, BRCA, KICH, and MESO. **B** KM plot showing that the expression of DIP2B predicted worse OS in BRCA, LUAD, MESO and KICH. Hexp: high expression; Lexp: low expression

The expression of DIP2B in 33 human cancers was analysed using TCGA and GTEx data sets. The results proved that the gene was highly expressed in 26 cancer types compared with normal tissues, in the following cancer types: ACC, BLCA, BRCA, CESC, CHOL, COAD, ESCA, GBM, HNSC, KICH, KIRC, KIRP, LAML, LGG, LIHC, LUAD, LUSC, OV, PAAD, PRAD, SKCM, STAD, TGCT, THCA, UCES and UCS (Fig. 1). The expression of DIP2B in different tumour cell lines in the CCLE expression profile is displayed in Supplemental Fig. 2, and the relationship between DIP2B expression and tumour stage is shown in Supplemental Fig. 3. The relationship between DIP2B expression and the OS of cancer patients was estimated. The results of Cox regression analysis indicated that the expression of DIP2B was closely related to poor OS in ACC, BRCA, KICH, and MESO (each *P* < 0.05; Fig. 2A). Furthermore, KM plot survival analysis confirmed that the high expression of DIP2B was associated with poor OS in BRCA, KICH, LUAD, MESO and THCA (each *P* < 0.05; Fig. 2B and Supplement Fig. 4). DIP2B expression was associated with poor OS according to both Cox analysis and KM survival analysis in BRCA, KICH and MESO; therefore, our further pancancer analysis of the DIP2B gene was focused on these three cancer types.

#### Pancancer analysis of the correlation between expression and immune infiltration

**Fig. 3:**
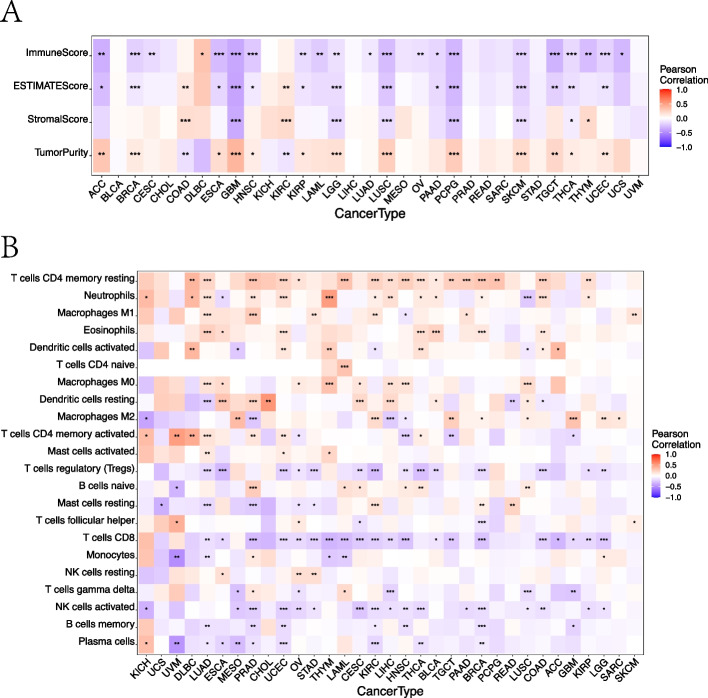
Relationship of immune cell infiltration and immune score with DIP2B expression across cancers. **A** The relationship between DIP2B expression and immune score across cancers. **B** The relationship between DIP2B expression and immune cell infiltration across cancers. *P < 0.05; **P < 0.01; ***P < 0.001

DIP2B showed a positive correlation with tumour purity and a negative correlation with immune score in most cancers, including BRCA (Fig. 3A). However, no significant relationship was found in KICH and MESO. Further analysis showed that the expression of DIP2B was positively correlated with infiltration of some immune cells, including neutrophils in 13 cancer types and macrophages M1 cells in 6 cancer types (Fig. 3B). The expression of DIP2B showed an inconsistent correlation with the different subtypes of CD4 + T cells (resting memory CD4 T cells, naive CD4 T cells, activated memory CD4 T cells, regulatory T cells and follicular helper T cells). However, DIP2B expression was negatively correlated with key immune killer cells, including CD8 T cells in 20 cancers, activated NK cells in 17 cancers, and plasma cells in 9 cancers (Fig. 3B). DIP2B expression showed a negative association with most of the immune killer cells in BRCA and some of the immune killer cells in MESO. No significant relationship with immune killer cell infiltration was found in KICH (Fig. 3B). The relationship between DIP2B and subclusters of immune cells across cancers is shown in Supplemental Fig. 5.

#### Pancancer analysis of the correlation between DIP2B and key immune regulatory genes

**Fig. 4:**
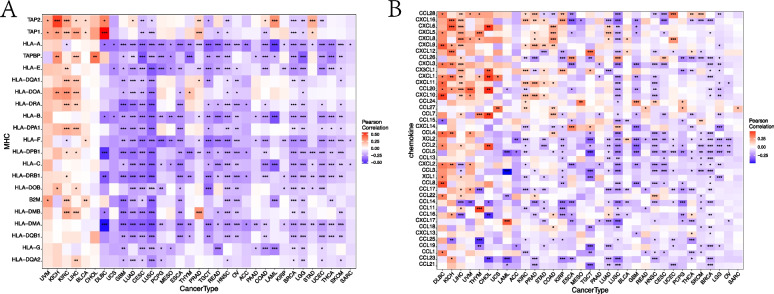
The correlation of DIP2B expression with major histocompatibility complex (MHC)-related genes (**A**) and chemokine-related genes (**B**) across cancers. *P < 0.05; **P < 0.01; ***P < 0.001

Gene coexpression analysis was further conducted to explore the relationship between DIP2B expression and tumour immune-related genes across cancers. DIP2B was correlated with different immune-related genes in different cancer types. DIP2B showed a significant negative correlation with major histocompatibility complex (MHC)-related genes and chemokine-related genes in BRCA (Fig. 4). In contrast, DIP2B showed a significant positive correlation with MHC-related genes and chemokine-related genes in KICH (Fig. 4). DIP2B expression did not affect the expression of MHC-related genes and chemokine-related genes in MESO (Fig. 4). Similar correlations between DIP2B and immunostimulator-related genes, immunoinhibitor-related genes and chemokine receptor-related genes were observed in KICH, MESO and BRCA (Supplemental Fig. 6).

#### Pancancer analysis of DIP2B expression and TMB and MSI

**Fig. 5:**
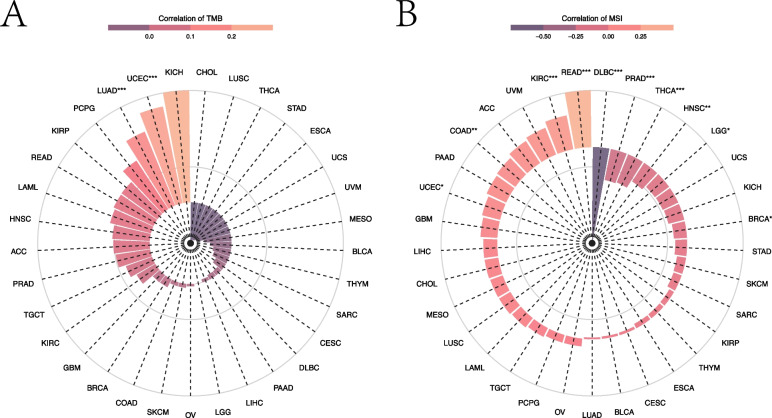
The relationship between DIP2B expression and tumour mutation burden (TMB) and microsatellite instability (MSI) across cancers. **A** Correlation between DIP2B expression and TMB across cancers. **B** The relationship between DIP2B expression and MSI across cancers. *P < 0.05; **P < 0.01; ***P < 0.001

TMB and MSI are two emerging biomarkers that can predict the response to immunotherapy. The results indicated that the expression level of DIP2B showed no significant correlation with TMB in KICH, MESO or BRCA (Fig. 5A). DIP2B only showed a slight negative correlation with MSI in BRCA (Fig. 5B).

### DIP2B analysis in BRCA subtypes

#### Expression and prognosis analysis in subtypes of BRCA

**Fig. 6:**
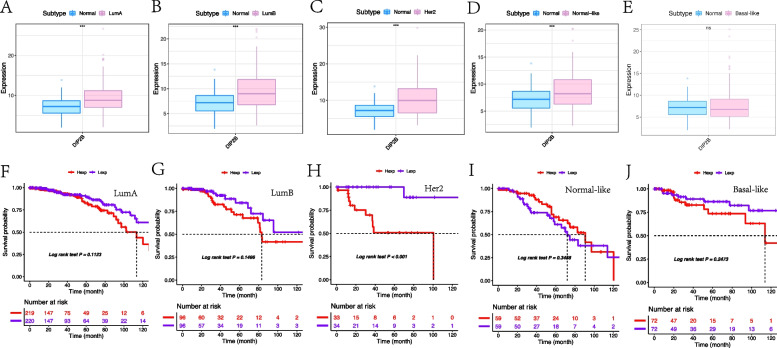
DIP2B expression and prognosis analysis in subtypes of BRCA. **A-E** The expression of DIP2B in subtypes of BRCA. **F-J** KM plot showing that the expression of DIP2B predicted worse OS in Her-2 + BRCA. Hexp: high expression; Lexp: low expression. ns = not significant (P > 0.05); *P < 0.05; **P < 0.01; ***P < 0.001

Five subtypes of BRCA (luminal A, luminal B, Her-2 + , normal-like and basal-like) were defined in the TCGA database based on gene expression profiling [18]. The expression of DIP2B in five subtypes of BRCA was analysed using TCGA and GTEx data sets. The results showed that DIP2B was highly expressed in the luminal A, luminal B, Her-2 + and normal-like subtypes (each *P* < 0.05; Fig. 6A-E). KM plot survival analysis showed that high expression of DIP2B was significantly associated with poor OS in the Her-2 + subtype (*P* < 0.05; Fig. 6F-J).

#### Analysis of expression, immune infiltration and key immune regulatory genes in subtypes of BRCA

**Fig. 7:**
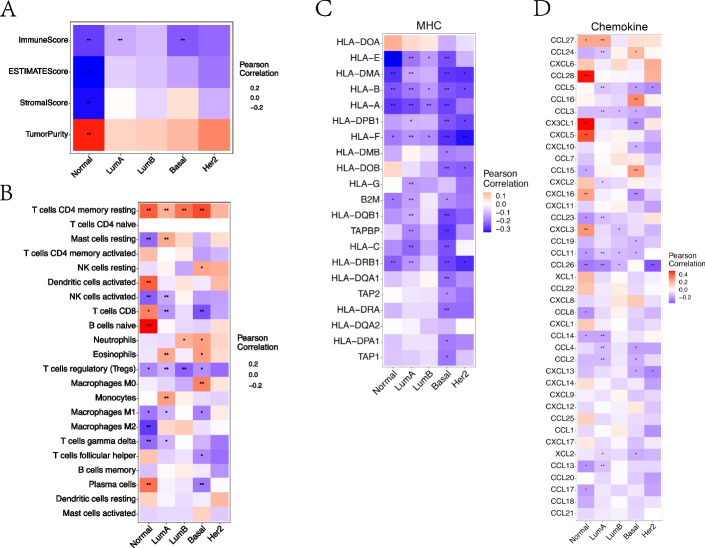
Correlation analysis of DIP2B expression and immune infiltration and the expression of key immune regulatory genes in subtypes of BRCA. **A** The relationship between DIP2B expression and immune score. **B** The relationship between DIP2B expression and immune cell infiltration. The correlations among DIP2B and major histocompatibility complex (MHC)-related genes (**C**) and chemokine-related genes (**D**). *P < 0.05; **P < 0.01; ***P < 0.001

DIP2B showed a positive correlation or tendency with tumour purity and a negative correlation or tendency with immune score in five subtypes of BRCA (Fig. 7A). Immune cell infiltration was further analysed in subtypes of BRCA. The infiltration of key killer immune cells was more strongly correlated with DIP2B in the normal-like subtype than in other types; specifically, DIP2B expression was associated with reduced activated NK cell and gamma delta T-cell infiltration and increased CD8 + T-cell and plasma cell infiltration (Fig. 7B). DIP2B expression in luminal A, luminal B, Her-2 + , and basal-like subtypes showed a consistent negative correlation with activated NK cell, gamma delta T cell, CD8 + T-cell and plasma cell infiltration (Fig. 7B). DIP2B expression showed a negative correlation with MHC-related genes and chemokine-related genes in subtypes of BRCA (except some chemokine-related genes in the normal-like subtype) (Fig. 7C-D). However, the correlation between the expression of DIP2B and that of immunostimulator-related genes, immunoinhibitor-related genes or chemokine receptor-related genes lacked consistency in BRCA subtypes (Supplement Fig. 7).

#### Expression and TMB and MSI analysis in subtypes of BRCA

**Fig. 8:**
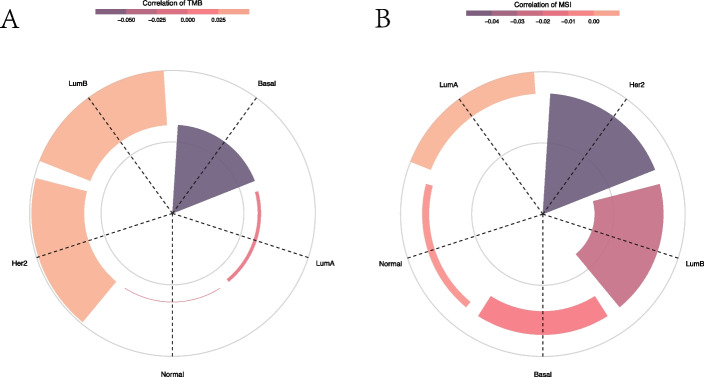
The relationship between DIP2B expression and tumour mutation burden (TMB) and microsatellite instability (MSI) in subtypes of BRCA. **A** Correlation between DIP2B expression and TMB. **B** The relationship between DIP2B expression and MSI. *P < 0.05; **P < 0.01; ***P < 0.001

The results indicated that the expression level of DIP2B showed no significant correlation with TMB and MSI in subtypes of BRCA (Fig. 8).

#### Expression analysis and GSVA in subtypes of BRCA

**Fig. 9:**
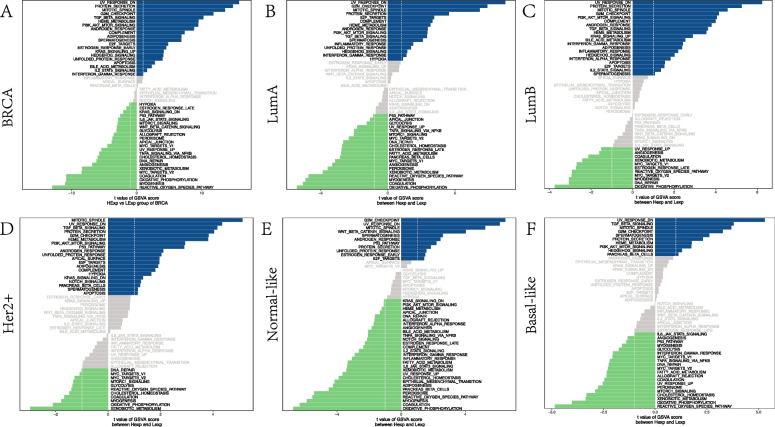
GSVA of genes related to DIP2B in BRCA (A). GSVA of genes related to DIP2B in the following BRCA subtypes: luminal A (**B**), luminal B (**C**), Her-2 + (**D**), normal-like (**E**) and basal-like (**F**). HExp: high expression; LExp: low expression

GSVA was first scored in BRCA, and then the samples were divided into high and low expression groups by DIP2B expression (Fig. 9). GSVA revealed that genes positively correlated with DIP2B were enriched in cancer-related pathways (PI3K-AKT) and proliferation-related pathways (MITOTIC_SPINDLE, G2M_CHECKPOINT and E2F_TARGETS) in BRCA (Fig. 9A). Genes negatively correlated with DIP2B were enriched in the DNA_REPAIR pathway in BRCA (Fig. 9A). Furthermore, GSVA of genes correlated with DIP2B was performed in each subtype of BRCA. Similar enrichment results were observed among BRCA subtypes (Fig. 9B-F).

#### Risk and prognosis analysis in BRCA according to the nomogram prediction model

**Fig. 10:**
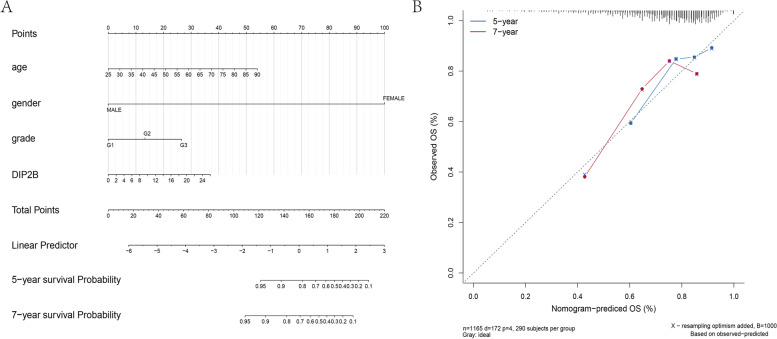
Construction of the nomogram model of DIP2B expression in BRCA. **A** DIP2B expression level for nomogram model prediction performance. **B** The correction curve of the nomogram model in the five-year and seven-year periods

According to DIP2B gene expression and clinical symptoms, a nomogram prediction model was constructed. The regression analysis results were displayed in the form of a nomogram. Logistic regression analysis showed that in BRCA samples, DIP2B gene expression showed high prediction efficiency as an important component of the model (Fig. 10A). Moreover, calibration curves for survival at 5 years and 7 years were generated, and the model predictions were consistent with the actual observations (Fig. 10B).

### Effect of DIP2B silencing in BRCA cell lines

**Fig. 11:**
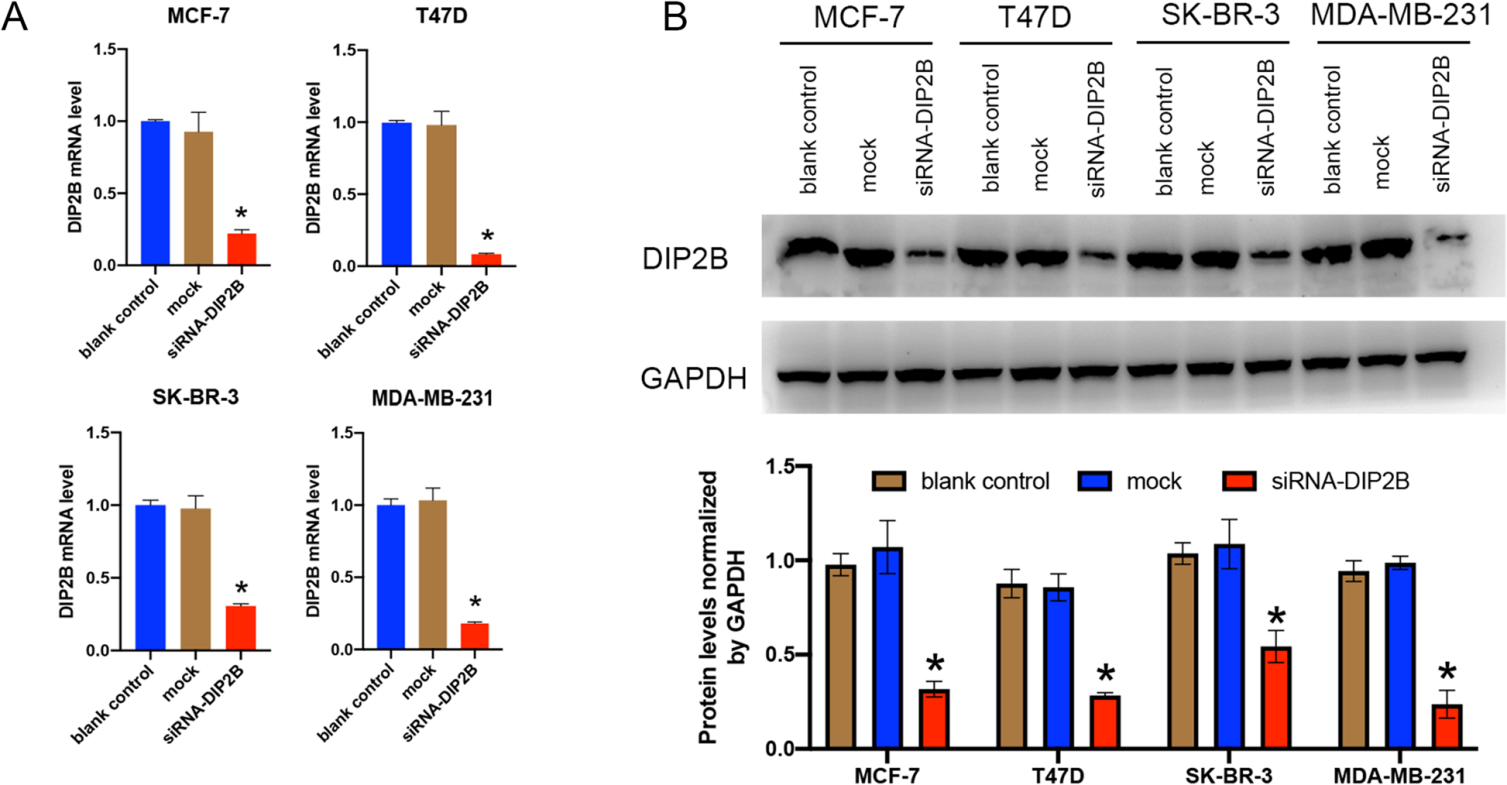
DIP2B silencing in breast cancer cells. Reduced DIP2B mRNA and protein levels in siRNA-DIP2B-transfected breast cancer cells were verified by real-time PCR and Western blotting. **A** Real-time PCR; the expression of DIP2B mRNA was normalized to that of GAPDH mRNA. **B** Western blotting; the expression of DIP2B protein was normalized to that of GAPDH protein. Results represent the mean ± SD of three independent experiments. *P < 0.05 vs. blank control group

After transfection with siRNA-DIP2B, quantitative real-time PCR assay was performed to analyse the expression of DIP2B mRNA. The expression of DIP2B mRNA in MCF-7, T47D, SK-BR-3 and MDA-MB-231 cells was suppressed in the groups transfected with siRNA-DIP2B compared with the blank control group and the mock group (each *P* < 0.05; Fig. 11A). We further assessed DIP2B protein expression in the same cell lines. DIP2B protein expression was significantly decreased in siRNA-DIP2B-transfected cell lines, according to Western blots (each *P* < 0.05; Fig. 11B). Original Western blots pictures were presented in Supplement Fig. 8. These results suggested that the expression of DIP2B was successfully reduced by siRNA-DIP2B in four breast cell lines.

#### DIP2B silencing suppressed breast cancer cell proliferation

**Fig. 12:**
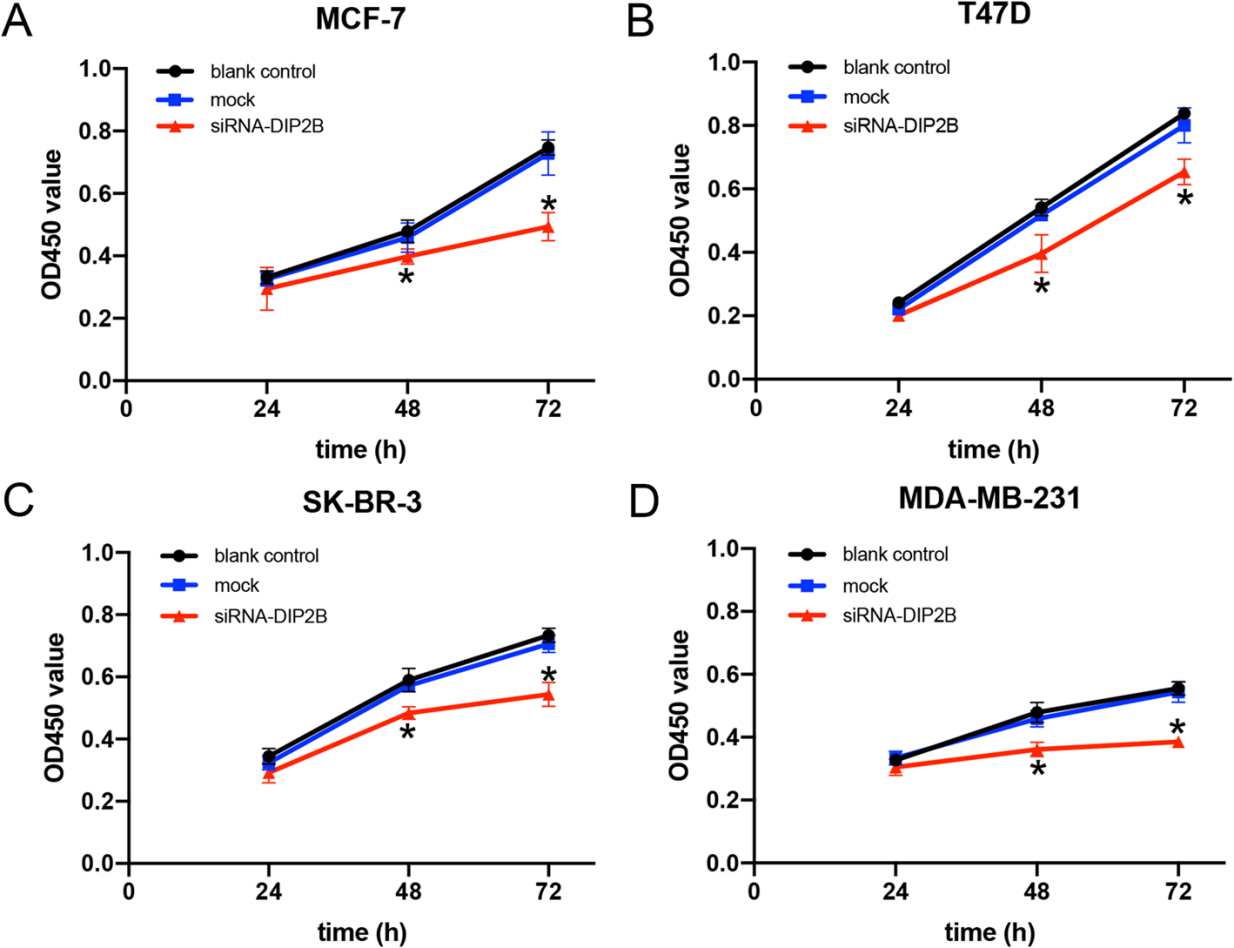
DIP2B-siRNA inhibits breast cancer cell proliferation. **A-D** Effects of DIP2B-siRNA on the cell proliferation were analysed with a CCK-8 assay and measured as OD values. Results represent the mean ± SD of three independent experiments. *P < 0.05 vs. blank control group at the same time

To analyse the role of DIP2B in the proliferation of breast cancer cells, we performed CCK-8 assays. Our data showed that the proliferation rates of four breast cell lines were significantly lower in the siRNA-DIP2B group than in the blank control group over a 72-h period starting at 48 h after transfection (each *P* < 0.05; Fig. 12). There were no significant differences between the blank control group and mock group. These results suggest that DIP2B inhibition significantly suppressed the proliferation of four breast cancer cell lines.

#### DIP2B silencing promotes apoptosis in breast cancer cells

**Fig. 13:**
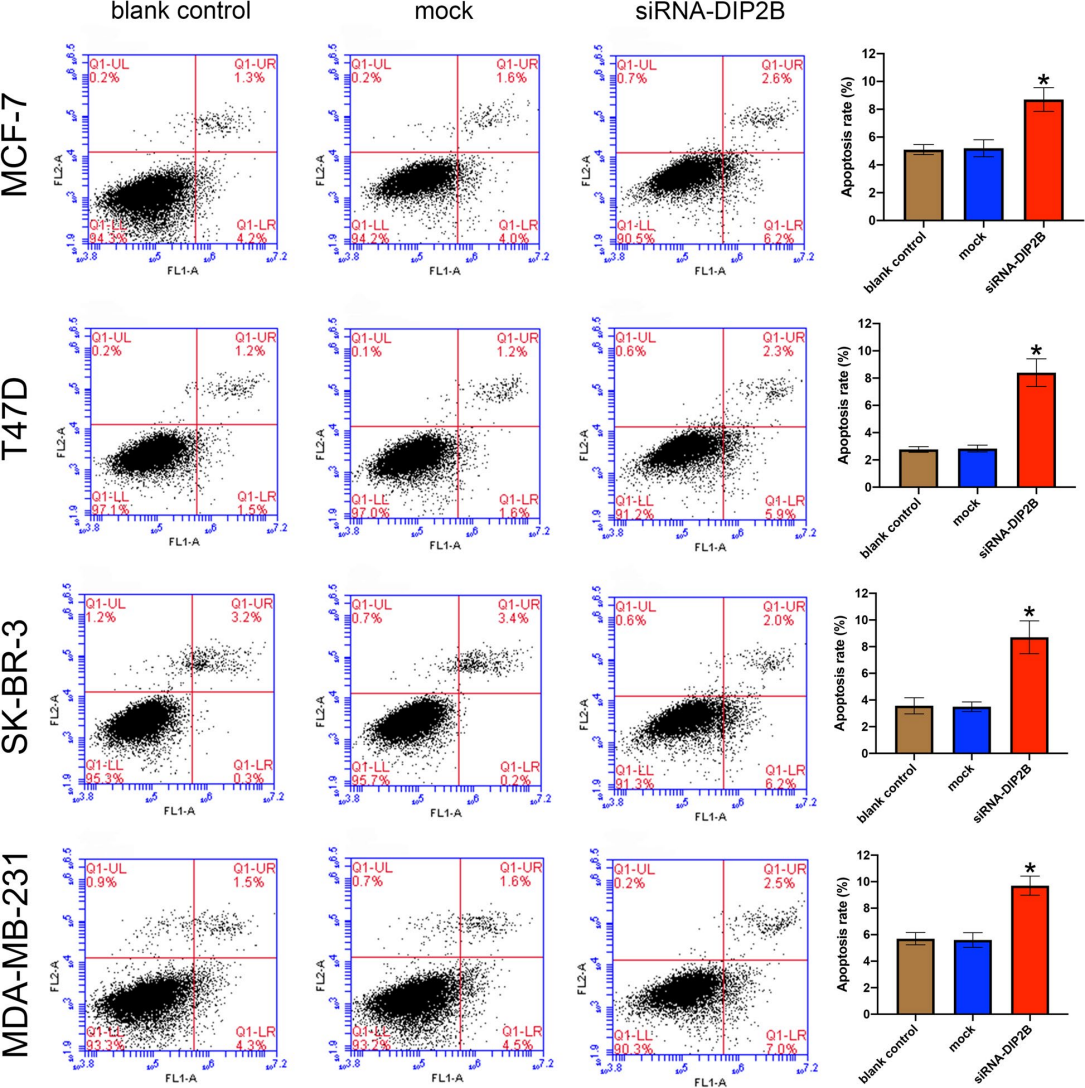
DIP2B-siRNA induces breast cancer cell apoptosis. Induction of apoptosis in breast cancer cells transfected with DIP2B-siRNA was analysed by flow cytometric analysis (Annexin V staining/propidium iodide). Results represent the mean ± SD of three independent experiments. *P < 0.05 vs. blank control group

To further confirm a potential role of DIP2B in apoptosis, we performed AV/PI staining by flow cytometry at 24 h post transfection. Our results showed that breast cancer cells transfected with siRNA-DIP2B had a significantly higher apoptosis rate than the blank control group (each *P* < 0.05; Fig. 13). There were no significant differences between the blank control group and the mock group. These results showed that siRNA-DIP2B induced cell apoptosis in breast cancer cells, which could also explain its ability to suppress breast cancer cell proliferation.

#### DIP2B silencing suppressed breast cancer cell migration

**Fig. 14:**
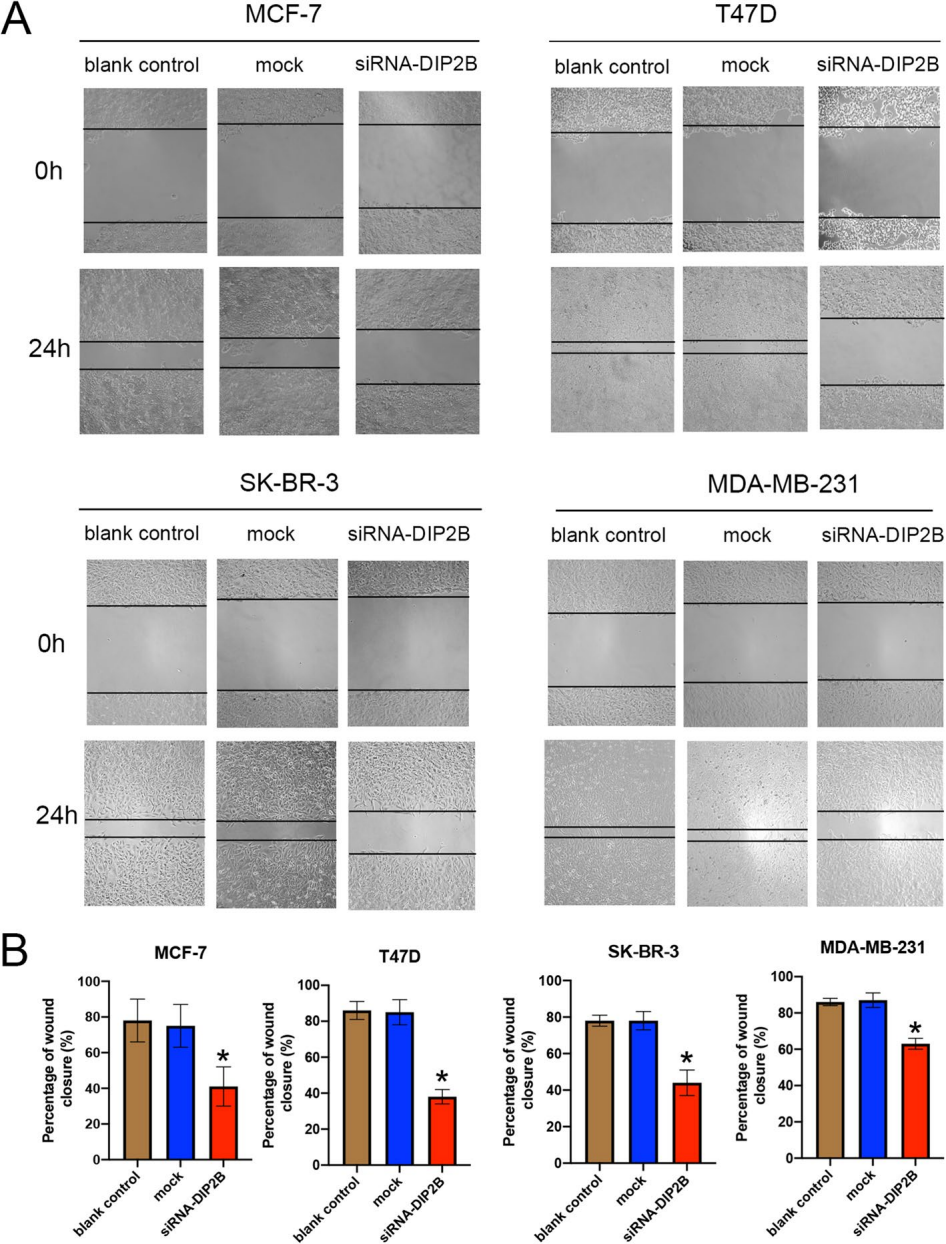
Effect of DIP2B siRNA transfection on the migration capability of breast cancer cell lines. **A** Representative wound-healing images at 0 and 24 h (magnification: × 100) in four breast cancer cell lines. The experiment was repeated three times independently. The scratch width had decreased significantly in the blank control and mock cultures after 24 h, whereas there was still a wider gap in the siRNA-DIP2B cells. **B** The percentages of wound closure were calculated. *P < 0.05 vs. blank control group

We investigated the effect of DIP2B silencing on the migration of the four breast cancer cell lines using a scratch assay. Confluent breast cancer cells were scratched, and a continuous cell-free region was observed. After 24 h, the scratch assay showed that the capacity of breast cancer cells to migrate into the ‘wound’ region was reduced in the group transfected with siRNA-DIP2B (Fig. 14A). The percentage of wound closure was significantly decreased in the siRNA-DIP2B group compared with the blank control group (each *P* < 0.05; Fig. 14B). No significant difference was observed between the blank control group and the mock group. These results showed that siRNA-DIP2B suppressed the migration capacity of breast cancer cells.

### Clinicopathological features related to DIP2B gene expression in BRCA

**Fig. 15:**
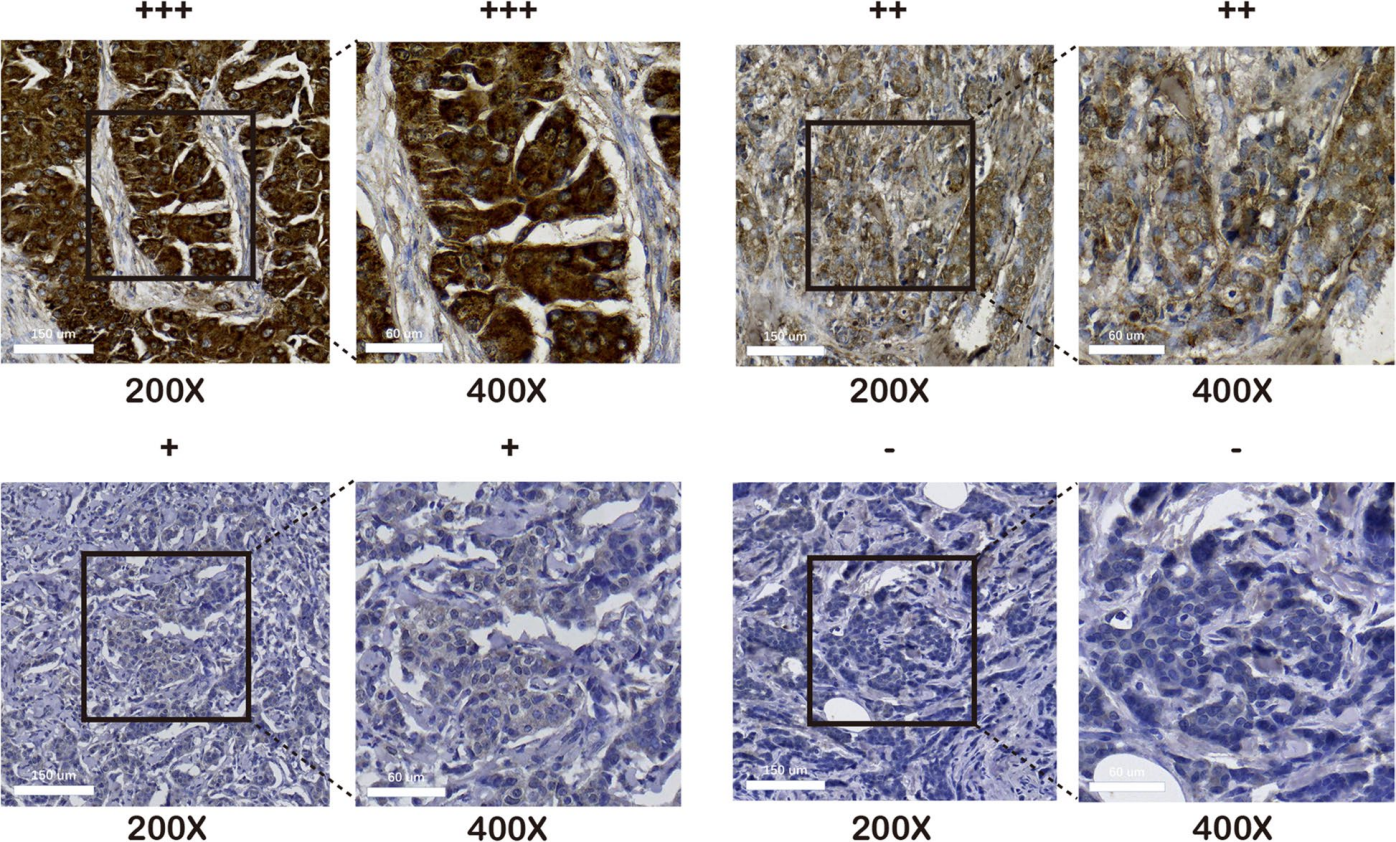
Representative images of positive and negative DIP2B protein expression in breast cancer tissues

**Fig. 16:**
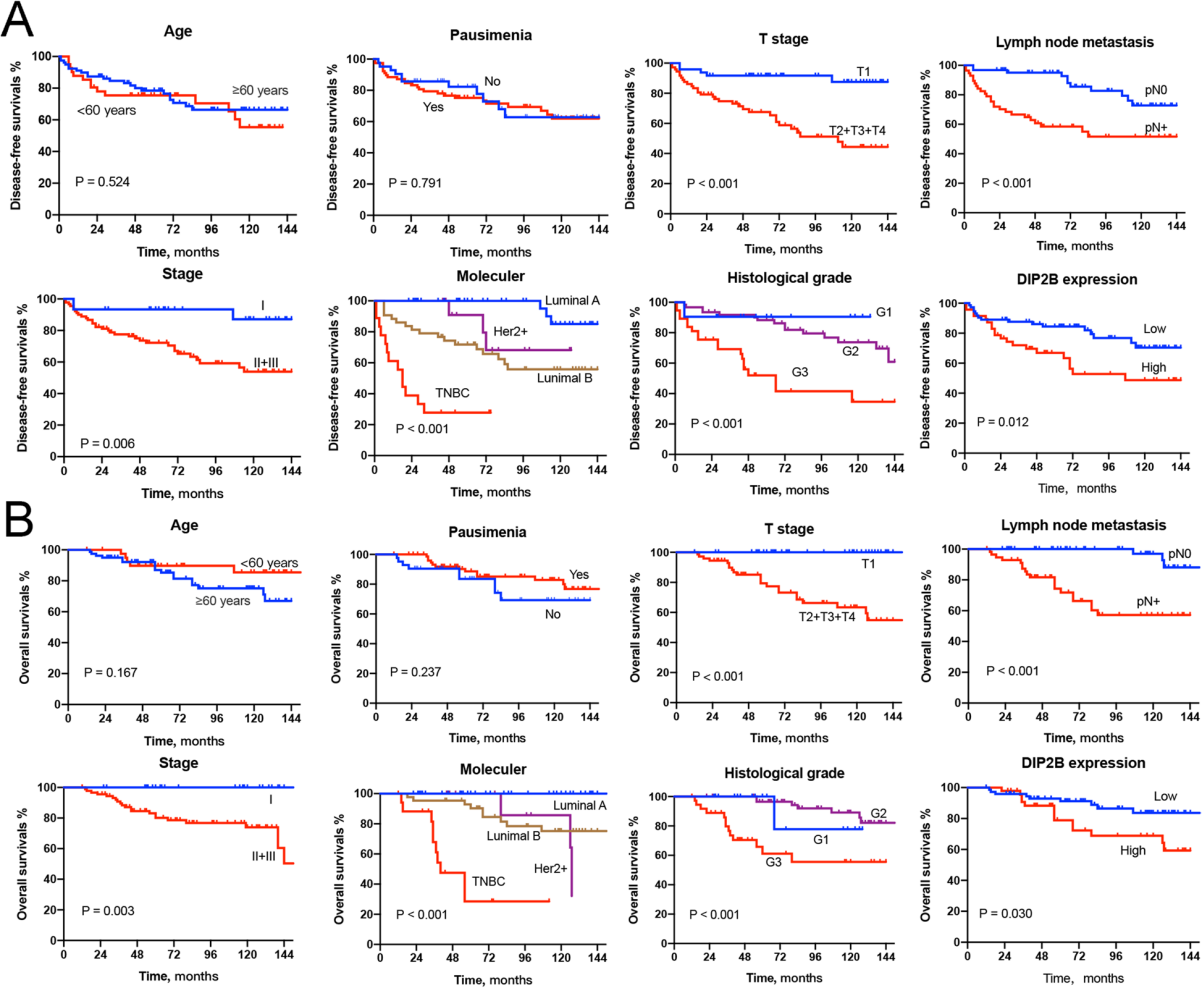
Kaplan‒Meier curves comparing (**A**) disease-free survival time and (**B**) overall survival time between groups of breast cancer patients

**Fig. 17:**
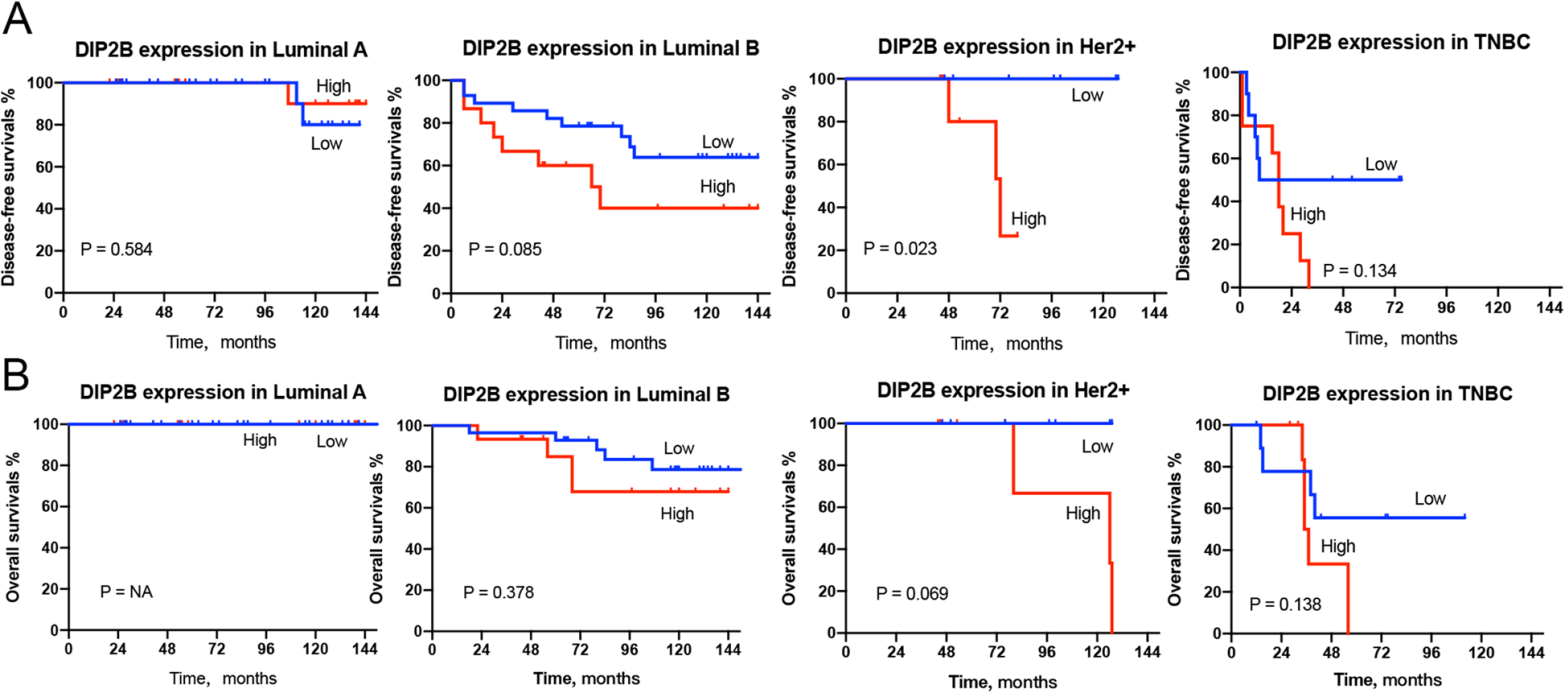
Kaplan‒Meier curves comparing (**A**) disease-free survival time and (**B**) OS time between DIP2B expression groups in subtypes of breast cancer patients

Immunohistochemistry was performed to detect DIP2B protein expression and subcellular localization in breast cancer tissues. The typical figures of DIP2B expression (from ‘-’ to ‘ +  +  + ’) are shown in Fig. 15. The results showed that DIP2B protein was expressed in the cytoplasm of breast cancer cells (Fig. 15). A total of 39.2% (47/120) of breast cancer samples showed high expression of DIP2B. Four subtypes of BRCA (luminal A, luminal B, Her-2 + and triple-negative breast cancer (TNBC)) were defined based on the expression of marker proteins [19]. High expression of DIP2B protein was significantly associated with high histological grade and lymph node metastasis (each *P* < 0.05; Table 1). Kaplan‒Meier analysis showed that T stage, N stage, TNM stage, molecular subtype, histological grade and expression of DIP2B were associated with disease-free survival and OS (each *P* < 0.05; Fig. 16). Univariate Cox regression analysis was performed for verification, and a similar result was observed, as shown in Table 2. Furthermore, Kaplan‒Meier analysis was performed in the subtypes of BRCA. The results showed that high expression of DIP2B tended to be associated with poor disease-free survival in luminal B BRCA (*P* = 0.085; Fig. 17) and predicted significantly reduced disease-free survival in Her-2 + BRCA (*P* = 0.023; Fig. 17). High expression of DIP2B was associated with a tendency towards poor OS in Her-2 + BRCA (*P* = 0.069; Fig. 17).

**Table 1:** Associations between DIP2B expression and clinicopathological parameters in breast cancer

Variable	DIP2B expression		*P value*
	Low^a^ (*n* = 73)	High^b^ (*n* = 47)	
Age, years (mean ± SD)	56.0 ± 10.0	56.8 ± 9.7	0.691
Menopause			
Yes	43	35	0.116
No	30	12	
T stage			
T1	32	16	0.342
T2 + T3 + T4	41	31	
Lymph node metastasis			
Yes	29	28	0.040
No	44	19	
Stage			
I	22	8	0.132
II + III	51	39	
Molecular subtype			
Luminal A	28	16	0.583
Luminal B	28	15	
Her-2 +	7	8	
TNBC	10	8	
Histological grade			
G1 + G2	58	25	0.004
G3	15	22	
Recurrence			
Yes	16	20	0.024
No	57	27	
Death			
Yes	9	13	0.052
No	64	34	

*SD* Standard deviation, *TNBC* Triple-negative breast cancer

^a^ − to +

^b^ +  + to +  +  +

**Table 2:** Associations between survival and clinicopathological parameters and DIP2B expression in breast cancer

Variables	DFS		OS	
	HR (95% CI)	*P value*	HR (95% CI)	*P value*
Age, years				
< 60	Reference	…	Reference	…
≥ 60	0.805(0.412–1.574)	0.526	1.989(0.733–5.402)	0.177
Menopause				
Yes	Reference	…	Reference	…
No	0.909(0.446–1.851)	0.792	1.664(0.707–3.914)	0.243
T stage				
T1	Reference	…	Reference	…
T2 + T3 + T4	0.184(0.071–0.475)	< 0.001	0.017(0.001–0.550)	0.021
Lymph node metastasis				
Yes	Reference	…	Reference	…
No	0.294(0.144–0.600)	0.001	0.108(0.032–0.365)	< 0.001
Stage				
I	Reference	…	Reference	…
II + III	0.178(0.054–0.581)	0.013	0.026(0.001–1.062)	0.075
Histological grade				
G3	Reference	…	Reference	…
G1 + G2	0.264(0.136–0.514)	< 0.001	0.194(0.082–0.457)	< 0.001
DIP2B expression				
Low^a^	Reference	…	Reference	
High^b^	0.441(0.228–0.853)	0.015	0.404(0.172–0.947)	0.037

*DFS* Disease-free survival, *OS* Overall survival, *HR* Hazard ratio, *TNBC* Triple negative breast cancer

^a^ − to +

^b^ +  + to +  +  +

## Discussion

The protein encoded by the DIP2B gene contains a binding site for transcriptional regulation by DNA methyltransferase 1-associated protein 1 and also contains AMP binding sites. The presence of these sites suggests that the DIP2B protein may take part in DNA methylation [2]. Hypermethylation of DNA CpG islands in the promoter region of key growth regulators (e.g., tumour suppressors) is a major pathway for the origin of many cancers [20]. However, there are few reports about the expression and role of DIP2B in tumours. Herein, we comprehensively and systematically investigated the role of DIP2B mRNA expression in 33 human cancers. We found that DIP2B was highly expressed in 26 cancer types compared with normal tissues. However, DIP2B expression was only associated with poor OS in BRCA, KICH, and MESO. Further analysis indicated that DIP2B expression was associated with poor OS in the Her-2 + subtype of BRCA based on the TCGA database. Furthermore, the prognostic value of DIP2B in BRCA was verified in a real-world clinical cohort by immunohistochemistry. Based on the results above, DIP2B might be considered a prognostic biomarker for BRCA, especially for the Her-2 + subtype.

DIP2B showed a positive correlation with tumour purity in most cancer types, which means that DIP2B is mainly expressed in cancer cells. Many studies have demonstrated that immune infiltration is associated with prognosis in cancers [21–23]. DIP2B showed a negative correlation with immune score in BRCA. CD8 + T cells, activated NK cells and plasma cells are considered key immune killer cells that can improve clinical outcomes and the response to immunotherapy treatment [24–26]. The analysis of immune cell infiltration showed that DIP2B had a negative correlation with key immune killer cells in BRCA. The analysis of immune-related genes indicated that high expression of DIP2B predicted low levels of MHC-related gene and chemokine-related gene expression in BRCA. MHC expression on tumour cells is important for the function of TILs, and downregulation of MHC might compromise the effective immune response in cancer patients. CD8 + T-cell recognition of tumour-specific peptides bound to MHC class I (MHC-I) molecules is central to tumour immunotherapy and tumour immunosurveillance [27]. Furthermore, increased MHC gene expression is associated with prolonged survival in most cancer types [28]. MHC gene expression, especially MHC class II expression, is associated with patient response to immune checkpoint blockade [28]. Immune cells are recruited and guided continuously into the tumour through interactions between chemokines and their receptors [29]. Therefore, the low level of immune infiltration might be derived from the low level of chemokine gene expression in the DIP2B high expression group in BRCA. DIP2B expression had no relationship with TMB and had a slight negative correlation with MSI in BRCA. Although the frequency of MSI in breast cancer is only 0–2%, MSI serves as a biomarker for checkpoint blockade response across tumour types. Based on the MSI results, high expression of DIP2B might predict a worse response to immune checkpoint blockade in BRCA. Because of the different gene expression profiles of the subtypes of BRCA, it is necessary to explore the tumour immune microenvironment of each subtype of BRCA separately. Interestingly, in different BRCA subtypes, the correlations between DIP2B expression and immune score, infiltration of key immune killer cells, and the expression of MHC-related genes and chemokine-related genes showed a consistent tendency. In different BRCA subtypes, high DIP2B expression was also associated with a low level of immune infiltration. These results indicated that high expression of DIP2B predicted a “cold” tumour immune microenvironment in BRCA.

We should also note that DIP2B showed different correlations with immune-related genes in KICH and MESO. In KICH, DIP2B expression showed a positive correlation with most of the immunoregulatory factor-related genes and even had a tendency to be related to TMB. We considered that DIP2B expression predicted a “hot” tumour immune microenvironment in KICH. In contrast to the results for DIP2B in BRCA and KICH, DIP2B expression was not associated with immune cell infiltration or the expression of immune-related genes in MESO, which means that DIP2B expression might have no correlation with the response to ICIs in MESO. Therefore, we considered that DIP2B might play different roles in different cancers. The implications of DIP2B expression in different cancers should be discussed separately and fully verified.

Furthermore, we revealed the role of DIP2B in cell signalling by GSVA in BRCA and its subtypes. We found that genes positively correlated with DIP2B were enriched in the PI3K-AKT pathway; when this pathway is overactive, as is the case in many cancers, apoptosis is suppressed and proliferation and migration are enhanced [30–33]. Genes coexpressed with DIP2B were also enriched in cell cycle-related pathways, including MITOTIC_SPINDLE, G2M_CHECKPOINT and E2F_TARGETS, which means that breast cancer cells with high expression of DIP2B showed stronger proliferative activity. In addition, genes negatively correlated with DIP2B were enriched in the DNA_REPAIR pathway. Defects in DNA repair pathways enable cancer cells to accumulate genomic alterations that contribute to their aggressive phenotype [34].

Immunohistochemical results of patient samples showed that the high DIP2B expression group of had an increased probability of lymph node metastasis, poor tumour differentiation and poor prognosis. We noted that both high mRNA expression and high protein expression of DIP2B were associated with poor prognosis in the Her-2 + breast cancer subtype, but the same was not true in other subtypes. However, we also observed consistently in all four classic breast cancer subtype cell lines that cell proliferation and migration capacity were significantly decreased after DIP2B knockdown in vitro, while the percentage of cell apoptosis was increased. We speculate that the inconsistency might be caused by the insufficient sample size in the prognosis analysis. However, the hypothesis needs to be further tested. Thus, what we have confirmed to date is that DIP2B is an oncogene in breast cancer, especially in the Her-2 + breast cancer subtype.

Promoting the immune infiltration of tumours with targeting agents might increase the response rate of ICIs [35, 36]. For example, PARP inhibitors or CDK4/6 inhibitors are promising targeting agents for combination with immunotherapy [37, 38]. We considered that DIP2B was also a promising therapeutic target. Inhibition of DIP2B might increase the immune infiltration of BRCA and improve the response to immunotherapy. However, Adlat et al. found that the function of immune cells was decreased in a mouse model with one allele knockout of DIP2B [15]. Therefore, specifically decreasing the expression of DIP2B in tumour cells is the first challenge. In addition, although DIP2B is known as a DNA methylation-related gene, the complete biological mechanism of DIP2B in different organs is still unclear, which means there is a risk of unpredictable complications. However, in the future, with an in-depth understanding of immunotherapy, treatments aimed at improving the tumour immune microenvironment are expected to become a new breakthrough.

### Limitations

Despite the fact that we conducted a thorough and systematic examination of DIP2B and employed many databases for multidimensional analysis, there are some limitations to our work. First, there is potential bias given the nature of bioinformatics analysis. Second, although 120 clinical samples were included, there is a potential bias due to an insufficient clinical sample size, especially in the analysis of BRCA subtypes. Third, data from in vivo models are lacking. Fourth, the specific mechanisms underlying the role of DIP2B in BRCA need to be further investigated.

In conclusion, DIP2B might be considered an oncogene and used as a prognostic biomarker in breast cancer. DIP2B expression predicts a cold tumour microenvironment, and DIP2B might serve as a potential target gene to improve the response of immunotherapy in breast cancer.

## Supplementary Information

**Additional file 1: sFigure 1.** The flow chart of the present study.

**Additional file 2: sFigure 2.** The expression of DIP2B in CCLE tumor cell line.

**Additional file 3: sFigure 3.** The relationship between expression of DIP2B and tumor stage in 19 cancer types.

**Additional file 4: sFigure 4.** The relationship between expression of DIP2B and overall survival in pancancer.

**Additional file 5: sFigure 5.** The relationship between expression of DIP2B and subtypes of immune infiltration cells in pancancer.

**Additional file 6: sFigure 6.** The correlation among DIP2B expression with immunostimulator related genes and immunoinhibitor related genes and chemokine receptor related genes in pan-cancer. *P ≤ 0.05; **P ≤ 0.01; ***P ≤ 0.001.

**Additional file 7: sFigure 7.** The correlation among DIP2B expression with immunostimulator related genes and immunoinhibitor related genes and chemokine receptor related genes in subtypes of BRCA. *P ≤ 0.05; **P ≤ 0.01; ***P ≤ 0.001.

**Additional file 8: sFigure 8.** Original Western blots pictures. A: DIP2B; B: GAPDH.

## Data Availability

Public data sets were acquired for this study. The databases and corresponding websites are as follows: TCGA (https://portal.gdc.cancer.gov/), GTEx (https://commonfund.nih.gov/GTEx), CCLE (https://portals.broadinstitute.org/ccle/), Xena (https://xena.ucsc.edu/), TISIDB (http://cis.hku.hk/TISIDB/), and Cellminer (discover.nci.nih.gov/cellminer).

## Abbreviations

ACC: Adrenocortical carcinoma
BLCA: Bladder urothelial carcinoma
BRCA: Breast invasive carcinoma
CESC: Cervical squamous cell carcinoma and endocervical adenocarcinoma
CHOL: Cholangiocarcinoma
COAD: Colon adenocarcinoma
DLBC: Lymphoid neoplasm diffuse large B-cell lymphoma
ESCA: Oesophageal carcinoma
GBM: Glioblastoma multiforme
HNSC: Head and neck squamous cell carcinoma
KICH: Kidney chromophobe
KIRC: Kidney renal clear cell carcinoma
KIRP: Kidney renal papillary cell carcinoma
LAML: Acute myeloid leukaemia
LGG: Brain lower grade glioma
LIHC: Liver hepatocellular carcinoma
LUAD: Lung adenocarcinoma
LUSC: Lung squamous cell carcinoma
MESO: Mesothelioma
OV: Ovarian serous cystadenocarcinoma
PAAD: Pancreatic adenocarcinoma
PCPG: Pheochromocytoma and paraganglioma
PRAD: Prostate adenocarcinoma
READ: Rectum adenocarcinoma
SARC: Sarcoma
SKCM: Skin cutaneous melanoma
STAD: Stomach adenocarcinoma
TGCT: Testicular germ cell tumour
THCA: Thyroid carcinoma
THYM: Thymoma
UCEC: Uterine adenocarcinoma
