# Supplementary figures and images for: Integrated pancancer analysis reveals the oncogene characteristics and prognostic value of DIP2B in breast cancer
Source: BMC Cancer. 2023 Mar 31;23:296. doi: 10.1186/s12885-023-10751-3 (PMC10064539; doi:10.1186/s12885-023-10751-3)

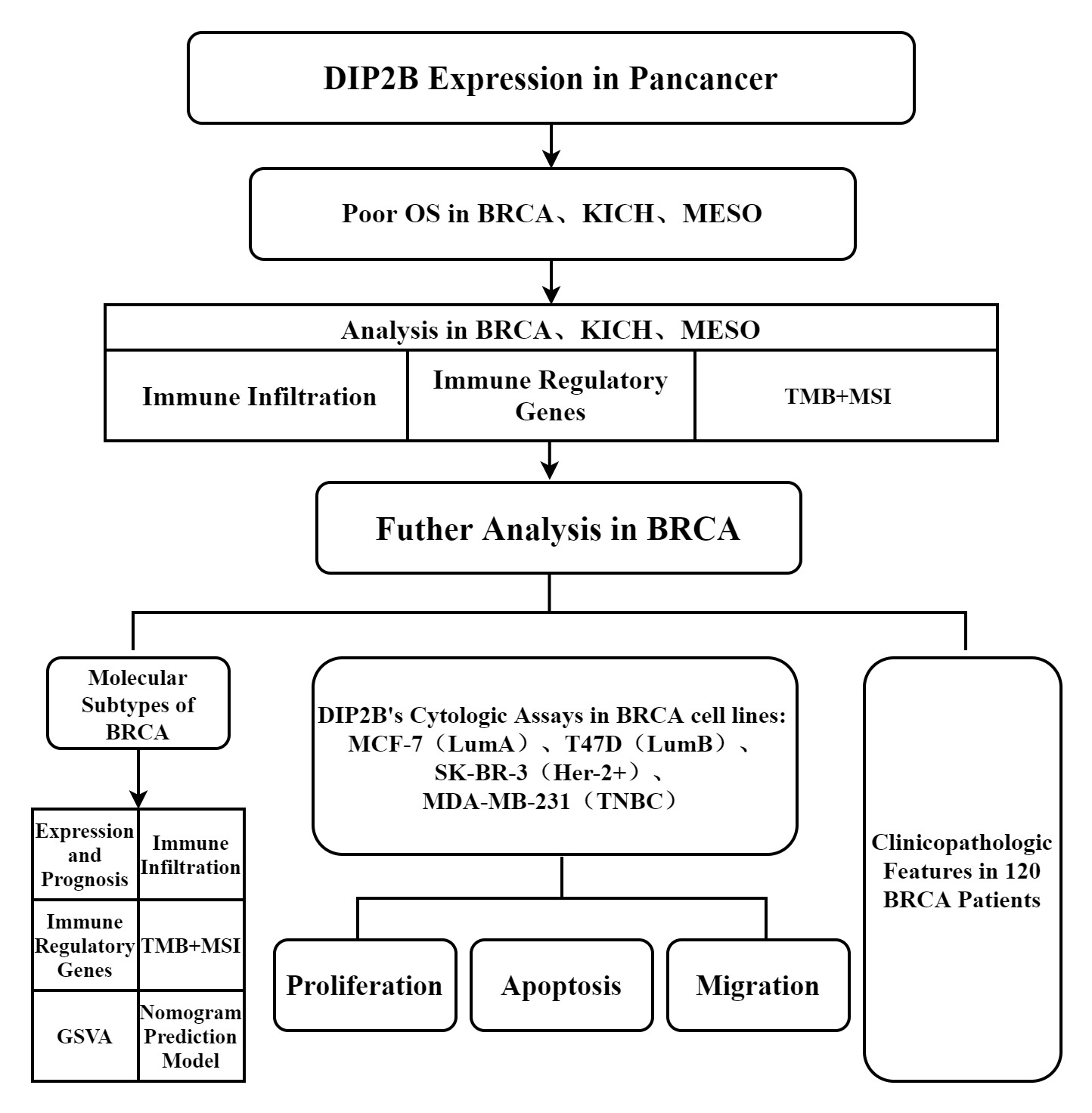

Supplement: Supplementary file 1 — Additional file 1: sFigure 1. The flow chart of the present study. [file 12885_2023_10751_MOESM1_ESM.jpeg]

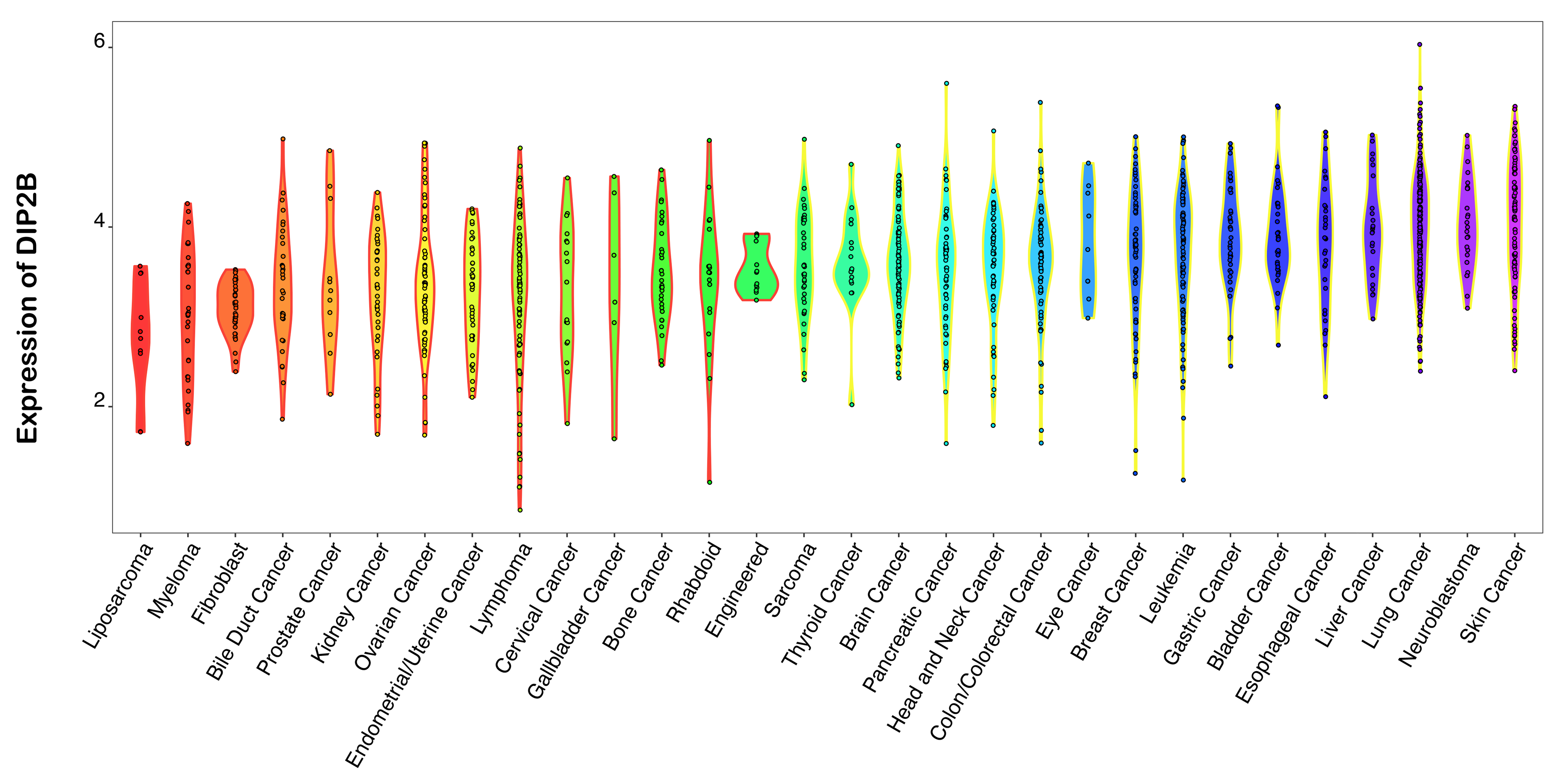

Supplement: Supplementary file 2 — Additional file 2: sFigure 2. The expression of DIP2B in CCLE tumor cell line. [file 12885_2023_10751_MOESM2_ESM.tif]

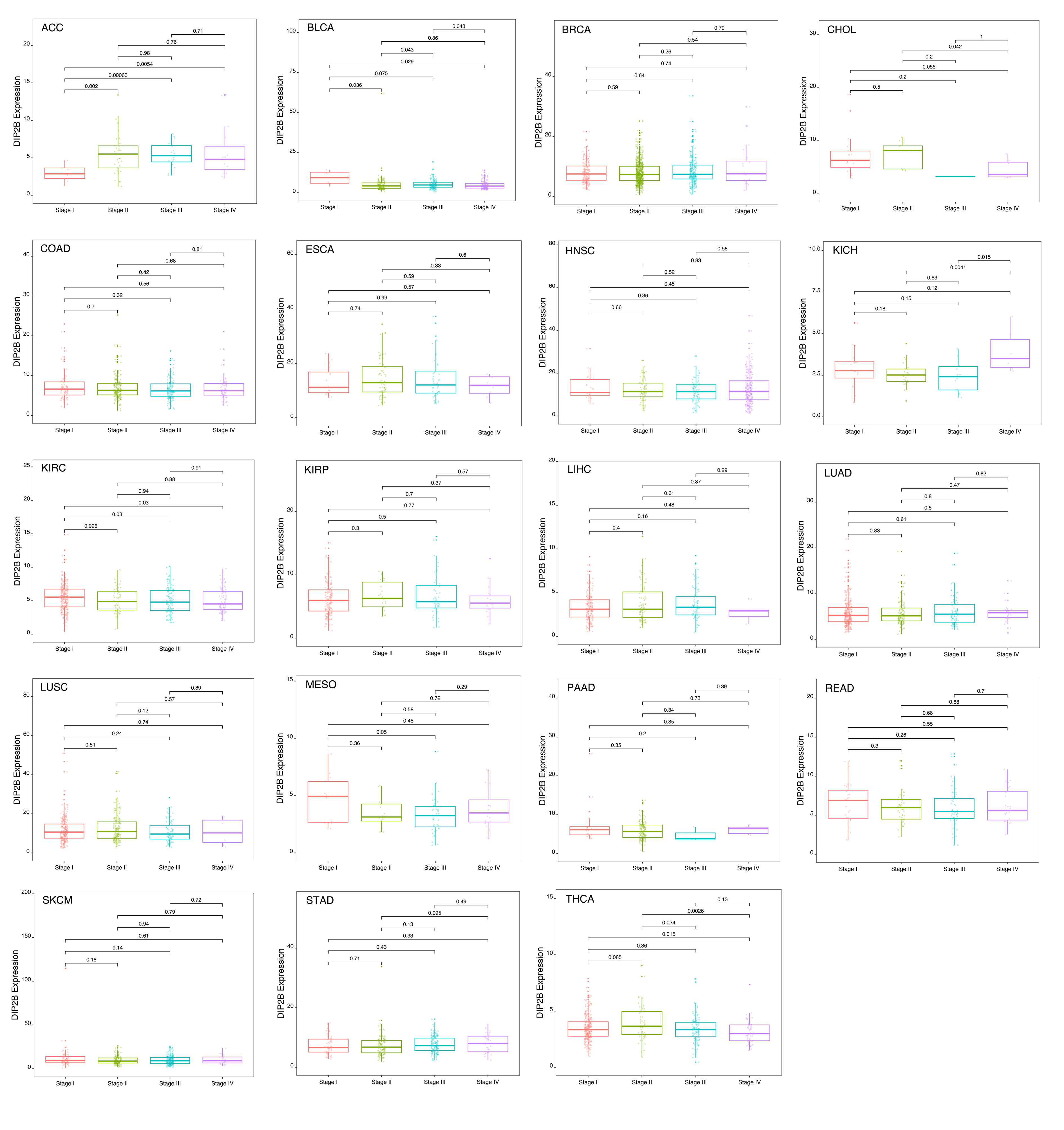

Supplement: Supplementary file 3 — Additional file 3: sFigure 3. The relationship between expression of DIP2B and tumor stage in 19 cancer types. [file 12885_2023_10751_MOESM3_ESM.tif]

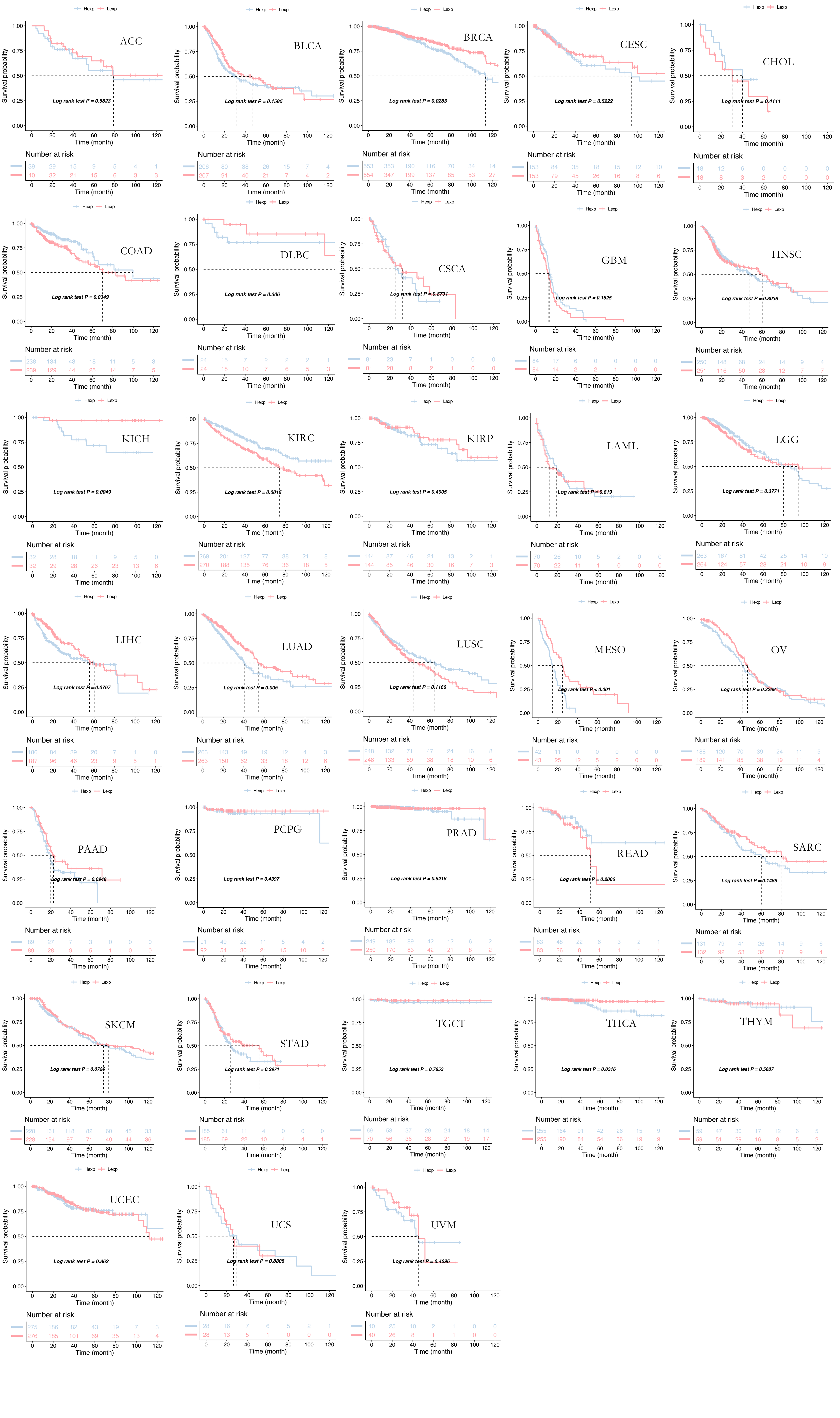

Supplement: Supplementary file 4 — Additional file 4: sFigure 4. The relationship between expression of DIP2B and overall survival in pancancer. [file 12885_2023_10751_MOESM4_ESM.tif]

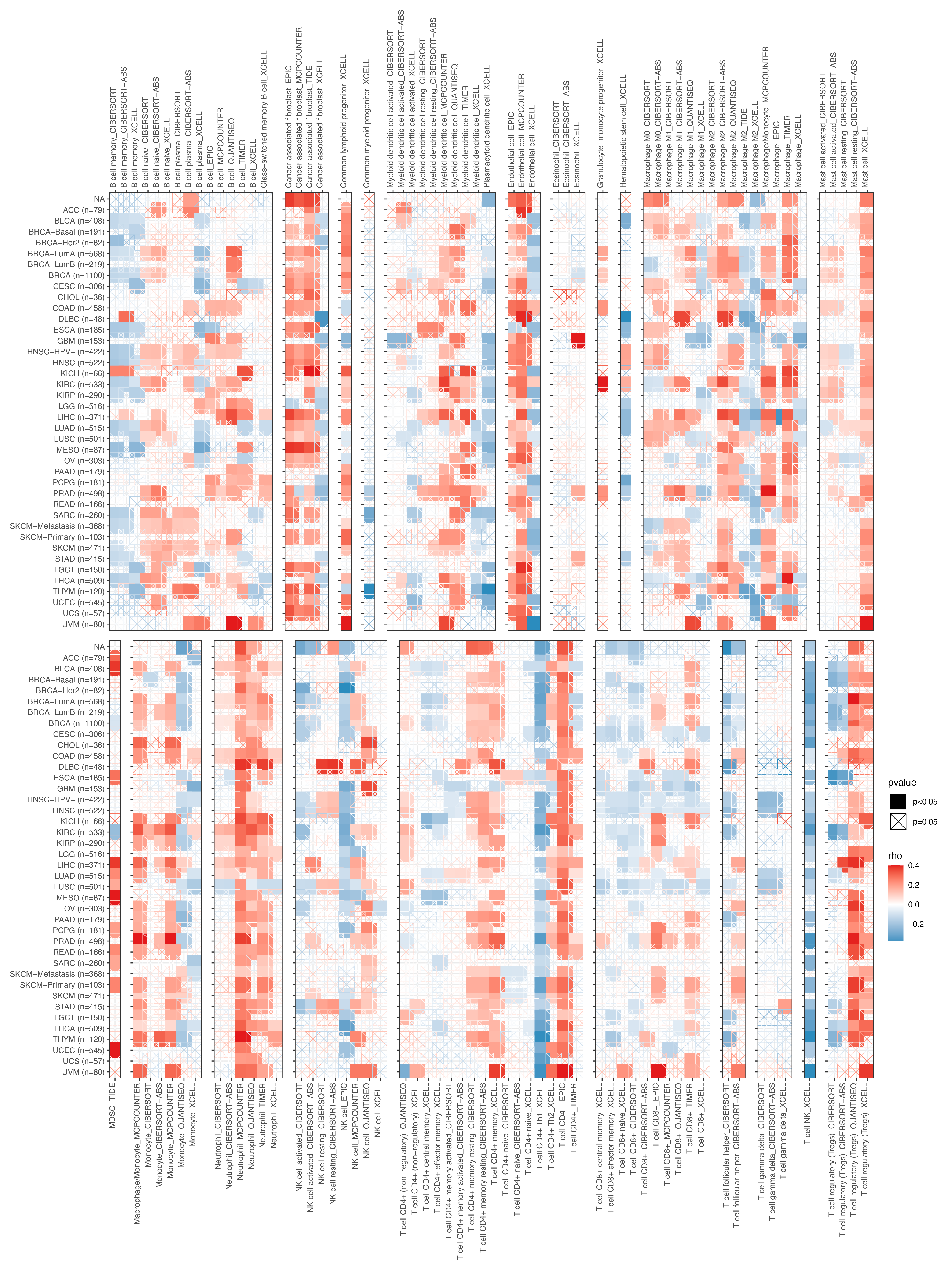

Supplement: Supplementary file 5 — Additional file 5: sFigure 5. The relationship between expression of DIP2B and subtypes of immune infiltration cells in pancancer. [file 12885_2023_10751_MOESM5_ESM.tif]

A

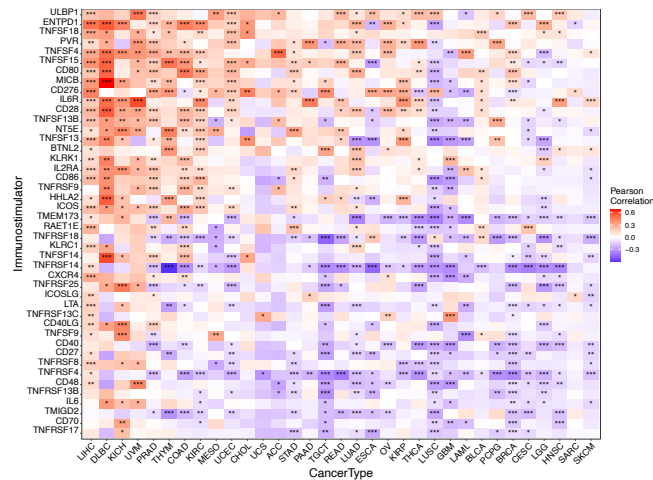

C

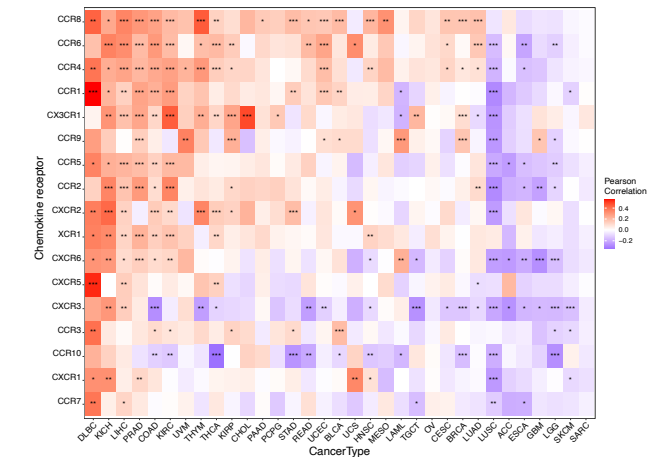

B

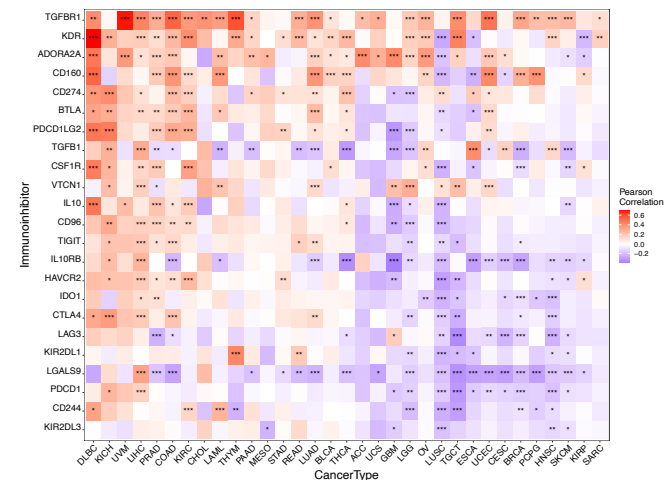

Supplement: Supplementary file 6 — Additional file 6: sFigure 6. The correlation among DIP2B expression with immunostimulator related genes and immunoinhibitor related genes and chemokine receptor related genes in pan-cancer. *P ≤ 0.05; **P ≤ 0.01; ***P ≤ 0.001. [file 12885_2023_10751_MOESM6_ESM.pdf]

A

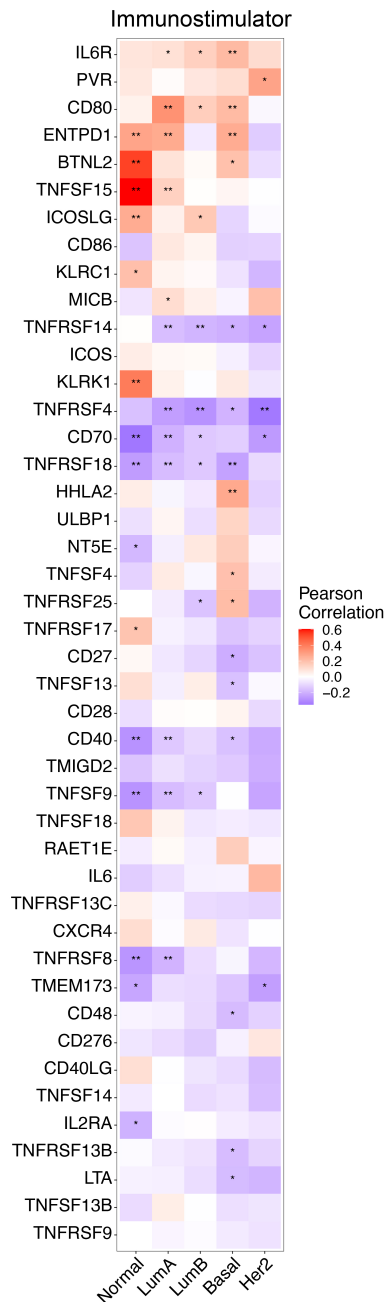

B

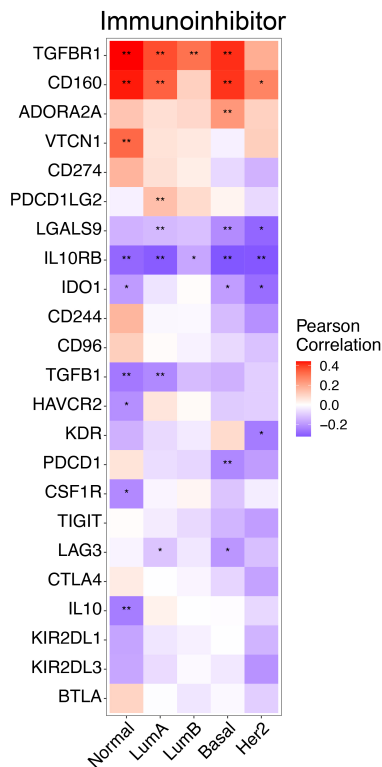

C

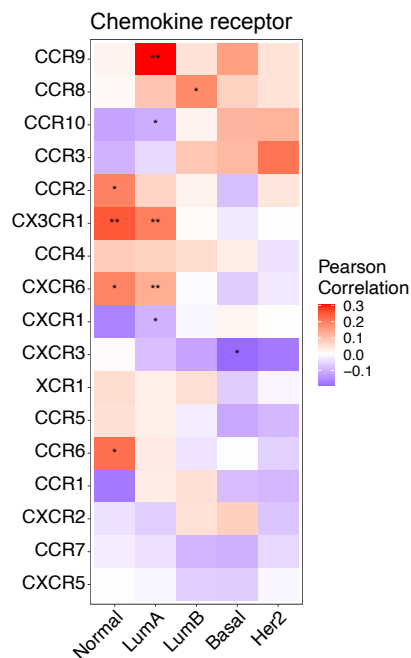

Supplement: Supplementary file 7 — Additional file 7: sFigure 7. The correlation among DIP2B expression with immunostimulator related genes and immunoinhibitor related genes and chemokine receptor related genes in subtypes of BRCA. *P ≤ 0.05; **P ≤ 0.01; ***P ≤ 0.001. [file 12885_2023_10751_MOESM7_ESM.pdf]

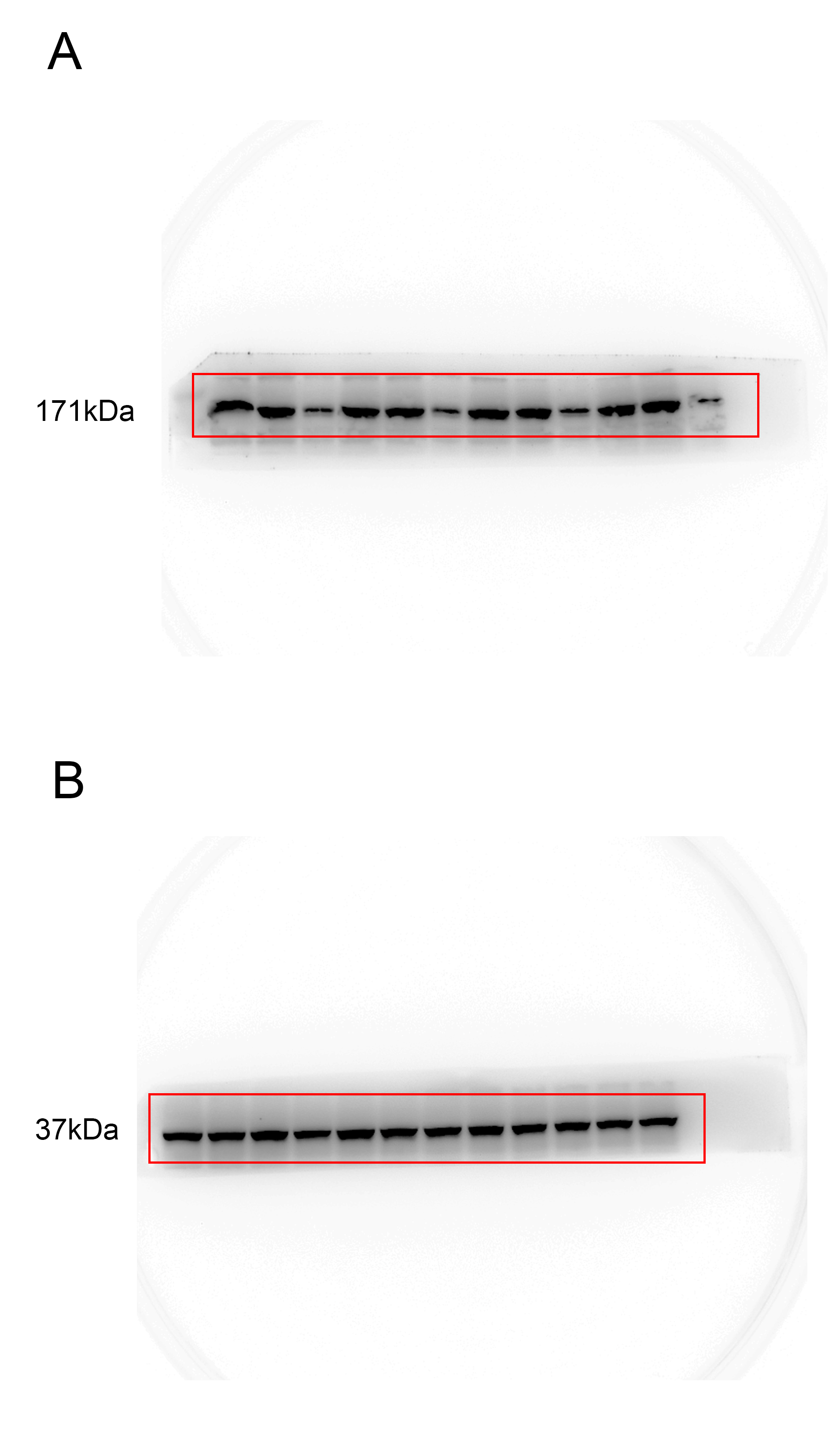

Supplement: Supplementary file 8 — Additional file 8: sFigure 8. Original Western blots pictures. A: DIP2B; B: GAPDH. [file 12885_2023_10751_MOESM8_ESM.tif]
